# MERRAclim, a high-resolution global dataset of remotely sensed bioclimatic variables for ecological modelling

**DOI:** 10.1038/sdata.2017.78

**Published:** 2017-06-20

**Authors:** Greta C. Vega, Luis R. Pertierra, Miguel Ángel Olalla-Tárraga

**Affiliations:** 1 Department of Biology and Geology, Physics and Inorganic Chemistry, Rey Juan Carlos University, Calle Tulipán s/n, Móstoles (Madrid) 28933, Spain

**Keywords:** Macroecology, Biogeography, Ecological modelling

## Abstract

Species Distribution Models (SDMs) combine information on the geographic occurrence of species with environmental layers to estimate distributional ranges and have been extensively implemented to answer a wide array of applied ecological questions. Unfortunately, most global datasets available to parameterize SDMs consist of spatially interpolated climate surfaces obtained from ground weather station data and have omitted the Antarctic continent, a landmass covering c. 20% of the Southern Hemisphere and increasingly showing biological effects of global change. Here we introduce MERRAclim, a global set of satellite-based bioclimatic variables including Antarctica for the first time. MERRAclim consists of three datasets of 19 bioclimatic variables that have been built for each of the last three decades (1980s, 1990s and 2000s) using hourly data of 2 m temperature and specific humidity. We provide MERRAclim at three spatial resolutions (10 arc-minutes, 5 arc-minutes and 2.5 arc-minutes). These reanalysed data are comparable to widely used datasets based on ground station interpolations, but allow extending their geographical reach and SDM building in previously uncovered regions of the globe.

## Background & Summary

The application of species distribution modelling (SDM) has boomed during the past ten years in the fields of biogeography, macro-ecology and conservation biology^1^. SDMs combine information on species occurrence with environmental characteristics to estimate the suitable distributional area^2^. The theory behind this relationship has been developed since the beginning of the 20th century^3^. From a macro-ecological perspective, climate-richness models based on water-energy dynamics^4^ have also displayed solid predictive ability to forecast responses to climate change (e.g., woody plants^5^). These models are built with environmental variables such as temperature and specific humidity, which are also physiologically meaningful^6,7,8, 9,10,11,12^ in different parts of the globe^13,14,15^. The advent of GIS and the increased availability of global environmental data in recent years have favoured the proliferation of diverse kinds of SDMs intended to answer a wide range of applied ecological questions^2^ (e.g., discovering biodiversity, conservation planning, health security, invasion ecology).

In the current macro-ecological research scene, WorldClim^16^ has become a most valuable and widely used source to retrieve high-resolution GIS climatic layers to build SDMs. These layers consist of spatially interpolated climate surfaces for global land areas obtained from weather station data using splines. WorldClim provides among other datasets 19 bioclimatic variables derived from precipitation and temperature records for the period 1950 to 2000. This set of bioclimatic variables describes temperature and water related annual tendencies, seasonality and extreme climatic conditions, including a combination of both environmental factors.

Despite the extensive application of WorldClim data in SDM approaches, some limitations have been recently identified as inherent to the usage of climatic datasets based on ground station interpolations^17^. While a high number of weather stations are spatially scattered to intensively survey the climatic conditions of highly urbanised countries, some large areas of the globe are not covered by a dense number of weather stations. For instance, some geographical areas at high latitudes and altitudes (such as Greenland), which are forecasted to undergo a dramatic temperature increase under current climate change scenarios^18,19^, lack direct climatic information from weather stations. Furthermore, a complete continental landmass as the Antarctic is omitted in WorldClim. Investigating the climate-driven redistribution of biodiversity in a warming planet would benefit from a detailed climatic description of these zones.

In parallel to the development and wide circulation of WorldClim, global-level satellite data collections have also become increasingly available and reanalyses of this information have served to deliver a set of physical and chemical variables to characterize the climatic conditions of the Earth’s surface^1^. These reanalyses combine a background forecast model and data assimilation routines. Then, the data assimilation fuses the available observations with the forecasts to produce uniform gridded data. Therefore, those areas accumulating more observation tools (grounded and remote) have higher accuracy levels, while those with low sampling effort are estimated using the forecast model. Remotely sensed information has improved the performance of SDMs^17^, including models aimed to assess the establishment of non-indigenous species in Antarctica^20,21^. In this context, the Modern Era Retrospective-analysis for Research and Applications (MERRA) is a NASA atmospheric data reanalysis of satellite information containing 28 data products with several variables each^22^.

**Figure 1:**
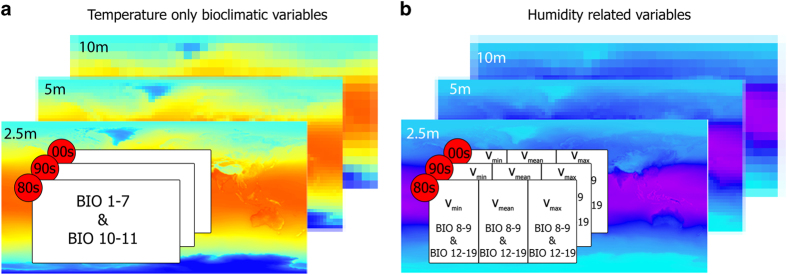
Structure of the MERRAclim dataset. (**a**) the temperature-only bioclimatic variables (BIO1-BIO7 & BIO10–11) are provided at 3 resolutions (coloured maps; 2.5 arc-minutes, 5 arc-minutes, 10 arc-minutes). For each resolution, a single dataset is available per decade (white boxes with red label; 1980s, 1990s, 2000s); (**b**) The humidity-related bioclimatic variables (BIO8–9 & BIO12–19) are provided at 3 resolutions (coloured maps). For each resolution three alternative versions are available (V_max_, V_mean_, V_min_) per decade (white boxes with red label).

Here, we have reproduced the computation and interpolation methods of WorldClim^23^ to generate MERRAclim, a global set of satellite-based bioclimatic variables. MERRAclim consists of three datasets of 19 bioclimatic variables that have been built for each of the last three decades using hourly data from the 1st of January 1981 to the 31st of December 2010. MERRAclim bioclimatic variables are computed from geographically homogeneous temperature and specific humidity gridded data and, hence, benefit from the same assimilation technique across the globe, including Antarctica. MERRAclim (Data Citation 1) datasets are derived from MERRA data, which has been extensively validated in the literature. We also provide a quantitative comparison of MERRA data and Antarctic Meteorological stations. The resolution of the gridded data has been done using a spline method to provide MERRAclim bioclimatic variables at three different resolutions (Fig. 1). We provide a comparison with WorldClim^16^ to facilitate the interpretation of future MERRAclim-based results with past research based on WorldClim.

## Methods

**Figure 2:**
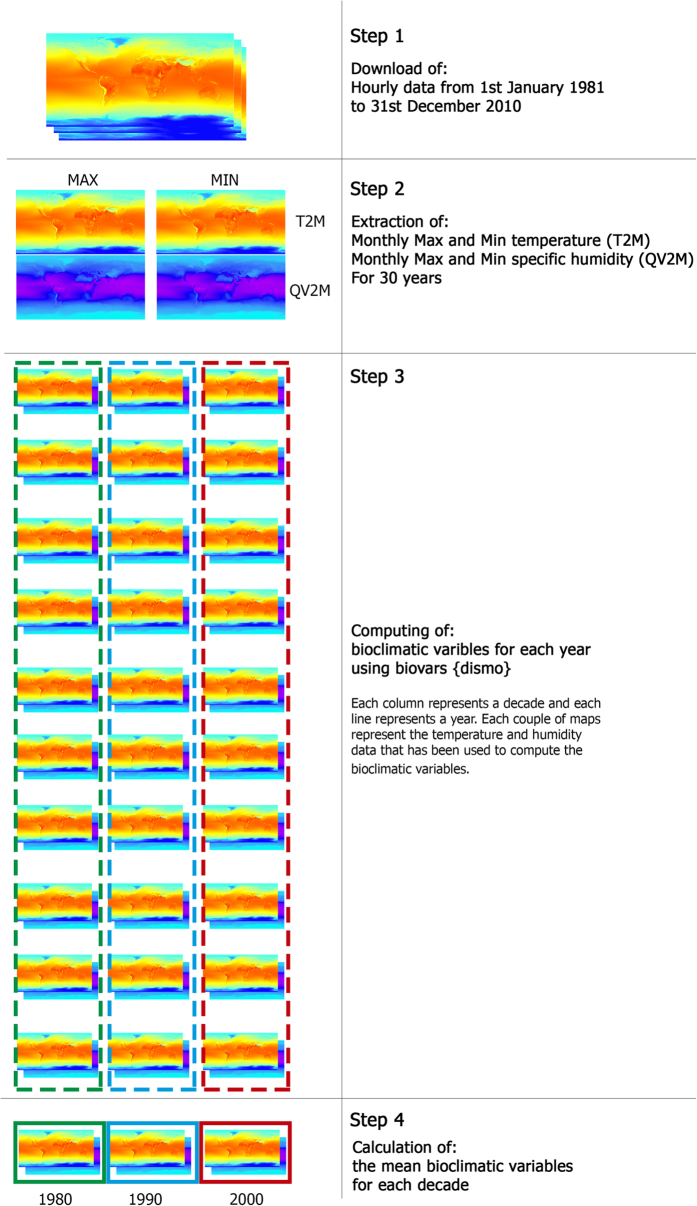
MERRAclim processing step by step. Computational steps followed to create the bioclimatic variables for each decade.

Step 1: We used 2 m air temperature (Kelvin degrees) and 2 m specific humidity (kg of water/kg of air) hourly data from the Modern Era Retrospective Analysis for Research and Applications Reanalysis^22^(MERRA) 2D Incremental Analysis Update atmospheric single-level diagnostics product (short name: MAT1NXSLV) provided by the NASA Global Modelling and Assimilation Office from the 1st of January 1981 to the 31st of December 2010 (Fig. 2). Specific humidity is an absolute measure of humidity which indicates the real amount of water present in the atmosphere that, contrarily to relative humidity, is not affected by changes in pressure or temperature^24^.

Step 2: After opening the downloaded NetCDF files using the R package *RNetCDF*^25^, for each month of the 30 year series, minimum and maximum temperature and specific humidity were extracted.

Step 3: For each year, three sets of bioclimatic variables were generated using the ‘biovars’ function of the R package *dismo*^26^. This function uses monthly minimum and maximum temperature and precipitation (mm) of the 12 months of a year following WorldClim protocols. Bioclimatic variables in WorldClim are: BIO1: Annual Mean Temperature, BIO2: Mean Diurnal Range, BIO3: Isothermality, BIO4: Temperature Seasonality, BIO5: Max Temperature of Warmest Month, BIO6: Min Temperature of Coldest Month, BIO7: Temperature Annual Range, BIO8: Mean Temperature of Wettest Quarter, BIO9: Mean Temperature of Driest Quarter, BIO10: Mean Temperature of Warmest Quarter, BIO11: Mean Temperature of Coldest Quarter, BIO12: Annual Precipitation, BIO13: Precipitation of Wettest Month, BIO14: Precipitation of Driest Month, BIO15: Precipitation Seasonality, BIO16: Precipitation of Wettest Quarter, BIO17: Precipitation of Driest Quarter, BIO18: Precipitation of Warmest Quarter and BIO19: Precipitation of Coldest Quarter.

For MERRAclim we used specific humidity (kg of water/kg of air) instead of precipitation (mm) values. To allow users’ choice of the most appropriate data for their ecological work we produced three versions of bioclimatic variables which depend on the specific humidity value used to produce them: a first one using monthly maximum specific humidity (V_max_), a second one using monthly mean specific humidity (V_mean_) and a third one using monthly minimum specific humidity (V_min_).

Step 4: Once the 30 datasets of bioclimatic variables (one for each year) and their respective three versions (V_max_, V_mean_, V_min_) were created, we merged them by calculating the mean for each decade (1980s, 1990s, 2000s) thus obtaining the following datasets: 80s(V_max_), 80s(V_mean_), 80s(V_min_); 90s(V_max_), 90s(V_mean_), 90s(V_min_); 00s(V_max_), 00s(V_mean_), 00s(V_min_). Spatial resolution of these datasets corresponds to the one of original MERRA raw data: 40 min of latitude and 30 min of longitude.

Step 5 (Fig. 1): Each dataset has been interpolated using the Spline geoprocess of type regularised, which yields a smooth surface and smooth first derivatives in ArcMap^27^, to obtain the datasets at the same three coarsest resolutions available in WorldClim (10 arc-minutes, 5 arc-minutes and 2.5 arc-minutes). Since its initial release to the public in 2005, WorldClim has been cited by 6,060 scientific papers, of which almost one fifth had a focus on SDMs (ISI Web of Science literature survey based on the search-string: TOPIC=’SDM’ OR ‘Species Distribution Model*’ OR ‘ENM’ OR ‘Environmental Niche Model*’; 19th of December 2016).

Spline is a deterministic interpolation method that has been shown to deliver similar results and sometimes slightly underperform when compared to Kriging^28,29,30,31^ (a stochastic method). Nevertheless, it has been commonly considered as appropriate for interpolation of densely sampled environmental variables^32^, for instance to produce WorldClim^16^, as it does not assume the process is normal nor stationary. Instead, the spline approach is based on the assumptions that the interpolation function passes through the data points and at the same time is as smooth as possible. This assumption is important as it implies that the data between two points that might be very different because of their physical characteristics will differ more depending on the interpolation technique used. Indeed, the absolute difference between the values obtained via Kriging and Spline of MERRAclim show that the littoral and high elevation areas have the larger bias that might reach 10 °C for BIO1 and 0.004 kg/kg for BIO12 (Supplementary Fig. 1).

Step 6: The final values have been multiplied by 10 for the temperature related variables (BIO1-BIO11) and by 100,000 the humidity related variables (BIO12-BIO19) to store the information as integers and therefore using rasters with a smaller depth of pixel allowing a faster download and easier manipulation in GIS software.

Step 7: As the biovars function was designed to be used with precipitation, not specific humidity, some of the resulting bioclimatic variables needed to be divided to have ecological meaning. Accordingly, the resulting BIO12 has been divided by 12 to obtain the final MERRAclim BIO12, which describes the annual mean of specific humidity instead of cumulative annual rainfall. The resulting BIO16, BIO17, BIO18 and BIO19 have all been divided by 3 so that the corresponding final MERRAclim variables inform on quarterly means instead of cumulative quarterly precipitation.

### Code availability

Code is available in Supplementary File 1.

## Data Records

**Figure 3:**
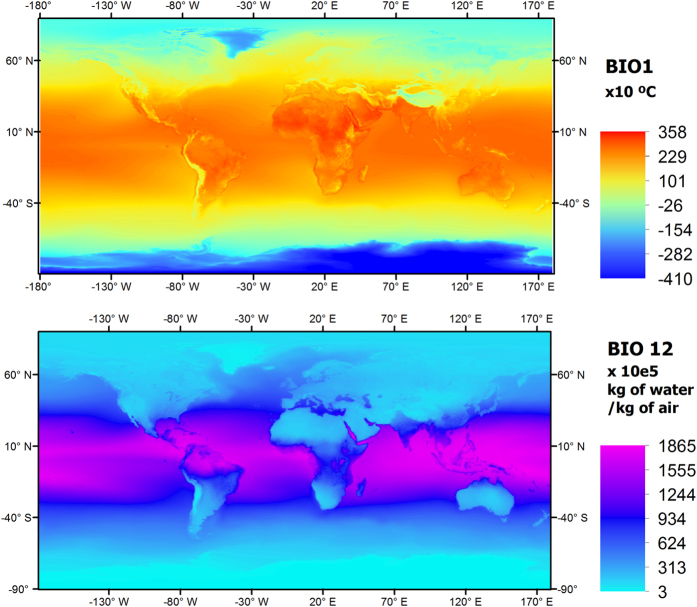
BIO1 (top; Annual mean temperature) and BIO2 (bottom; Annual mean humidity) from the 2000s decade V_min_ at 10 arc-minutes resolution.

The MERRAclim dataset (Data Citation 1,Fig. 1) is provided for three decades (1980s, 1990s and 2000s) in three versions (V_min_, V_mean_ and V_max_) and at three spatial resolutions (10 arc-minutes, 5 arc-minutes and 2.5 arc-minutes). We provide users with the three versions so they can choose the one that best meets their research needs. Example layers for BIO1 and BIO12 from the 2000s decade V_min_ at 10 arc-minutes resolution are depicted in Fig. 3. The datasets are zipped folders and are named following the convention: resolution_version_decade. Each folder contains the 19 bioclimatic variables (Table 1) as georeferenced GEOtiff files and are titled with the standard combination: resolution_version_decade_bioclimatic.tif. BIO1- BIO11 represent temperature (in degree Celsius multiplied by 10) and BIO12-BIO19 are specific humidity (kg of water/kg of air multiplied by 100,000). Each of these zip folders can be downloaded individually.

Temperature-related bioclimatic variables (BIO1-BIO7 and BIO10-BIO11) are identical in the three versions of the dataset because they do not rely on specific humidity data which is the variable that is inputted in three different versions (see Methods). The remaining bioclimatic variables show very little variation among the three different versions (see Usage Notes).

MERRAclim is derived from MERRA, a global reanalysis that assimilates available ground and satellite observations with a background model forecast. Thus, its uncertainty, as a reanalysis, is related to the location of in situ and remote observations. Consequently, developed nations in the Northern Hemisphere have smaller uncertainty than isolated areas^33^. MERRA has been evaluated and compared to other reanalyses since its release, we refer to the literature in the Technical Validation to justify its suitability to derive bioclimatic variables. In addition to this, we have performed a quantitative comparison between MERRA and Antarctic ground stations which shows a strong correlation although the values from MERRA are colder.

We provide a comparison between MERRAclim and WorldClim (see Usage Notes) to assist with the choice of version. Water-related variables (BIO12-BIO17) and the combined bioclimatic variables (BIO8, BIO18 and BIO19) from version V_min_ are the ones that correlate the most with their corresponding bioclimatic variables from WorldClim, whereas BIO9 correlates more strongly with its WorldClim counterpart from V_max_ or V_mean_. Overall, MERRAclim varies the most compared to WorldClim for those bioclimatic variables sensitive to extremes.

## Technical Validation

The Modern-Era Retrospective Analysis for Research and Applications (MERRA) was made public in 2011 aiming to improve upon the hydrologic cycle described in earlier reanalyses^22^. We chose MERRA to produce global bioclimatic variables as, in several evaluations englobing several decades, it showed high reliability for water^34^ and energy variables^35^ at different scales and in different regions. To reinforce these validations, we have carried on a quantitative comparison between MERRA and Antarctic ground stations.

At large scales, the comparisons with other reanalyses showed that MERRA performed similarly^36,37^ or outperformed some^38,39^ (Table 2). Although it presents some weaknesses these have also been found in past reanalyses^40^. Regionally, MERRA has been compared for Polar Regions where it has shown to be one of the most consistent for both water^41,42^ and energy variables^43,44^. Although it has been demonstrated that it contains some errors for the energy budgets, these are not directly related with temperature^45^.

At more regional scales, the comparisons between reanalyses show that each modern reanalyses perform better from one area to another, for instance, in Alaska, MERRA is the best reanalysis for interior areas, while other reanalyses are more suitable in North and the South-eastern Alaska^46^.

**Figure 4:**
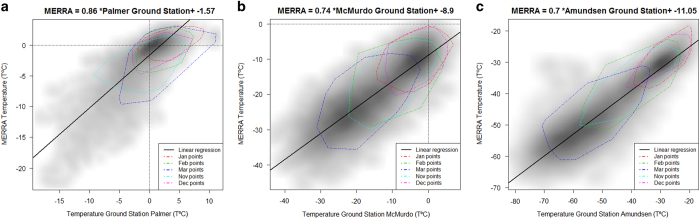
Density scatterplot of temperature time series for MERRA and United States Antarctic Program meteorological stations in (a) Palmer (2007–10), (b) McMurdo and (c) Amundsen-Scott South Pole. Darker grey represents a higher density of points. The dashed polygons represent the distribution of the points for the warmest months: January (red), February (green), March (dark blue), November (light blue) and December (pink). The estimated parameters of the fitted linear relationship are at the top.

**Figure 5:**
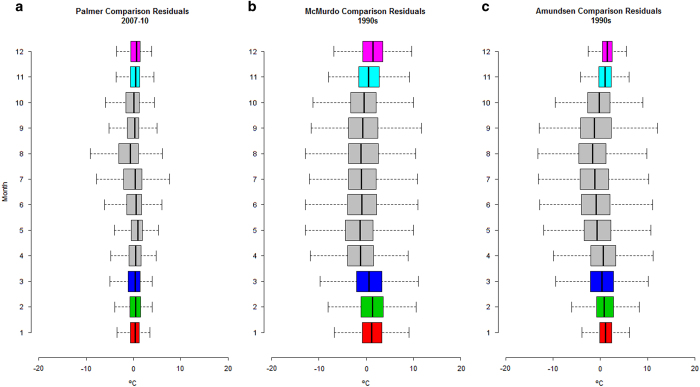
Distribution of the residuals of the linear models for MERRA and United States Antarctic Program meteorological stations in (a) Palmer (2007–10), (b) McMurdo and (c) Amundsen-Scott South Pole by month.

We have compiled hourly data from the University of Wisconsin-Madison Automatic Weather Station Program (http://amrc.ssec.wisc.edu) to estimate the regional variability, the correlation and the bias of MERRA data in Antarctica. We used temperature time series for three United States Antarctic Program (USAP) bases each located in a different Antarctic region: Palmer (West Antarctica, 2007–2010, data not available from June to November 2010), McMurdo (East Antarctica, 1990s decade) and Amundsen-Scott South Pole (Interior Antarctica, 1990s decade) (Table 3). For Palmer and Amundsen time series the data is available hourly, whereas for McMurdo the time series step is every 6 h. Pearson’s correlation coefficients for the three regions show a high correlation (over 0.8) between MERRA and USAP ground stations, being stronger for higher latitudes (Table 3). The same relationship trend is shown by the linear regressions (with a slope between 0.69 and 0.86) that explained over 68% of the variance and showed that MERRA records were colder, with the largest difference in Amundsen (11 °C) (Fig. 4). However, the residuals of the linear regressions show that bias over 5 °C are rare (Fig. 5). Indeed, Inter Quartile Range of the residuals are between 2.4 and 5.4 °C (Table 3) and are larger for the coldest months when the low temperature does not allow ecological activity (Fig. 5). Furthermore, we have compared the resulting bioclimatic variables using both datasets (Table 4), this comparison leads to the same conclusion as the hourly comparison: MERRA records are colder than USAP records but, as they are summarised values, the difference is smaller than when comparing hourly.

Everything considered, MERRA data is one of the best reanalyses available with a global extent as the evaluations at different scales in different regions have shown. In the Antarctic region in particular, the temperatures recorded by MERRA are colder than the ground stations, but this bias is small during the summer months, when the biological activity takes place and it is more visible in extremely high latitudes, i.e., the South Pole, where ecologically viable temperatures are never reached.

## Usage Notes

### Comparison between MERRAclim (80s and 90s decades) and WorldClim

MERRAclim datasets were created using temperature and specific humidity and following the methods described for WorldClim^23^, to derive 19 bioclimatic variables that can be used in ecology. This section provides a comparison of MERRAclim and WorldClim to find possible patterns of spatial congruence or discordance. We calculated Pearson’s correlation coefficients and fitted linear regressions to assess the relationship between both datasets^47^.

The comparison is geographically limited to those areas where WorldClim data are not interpolated, i.e., around weather stations that were used to compile information. As WorldClim is temporally limited to climatic records ranging from 1950 to 2000, the MERRAclim datasets could only be compared for the 80s and 90s decades.

### Framing the geographical extent for the comparison WorldClim vs. MERRAclim

MERRA raw data is composed of a grid made of 540 columns and 360 rows. Each grid cell covers 2/3 degrees of latitude and < degrees of longitude, i.e., each cell covers an area of 1/3 square degrees. To make both datasets comparable we geographically limited the WorldClim dataset by creating an area of influence around each weather station as a buffer zone that covers 1/3 square degrees (equivalent to an area of ≈ 4,000 km^2^ near the Equator and ≈250,000 km^2^ for the farthest north weather station included in WorldClim at a latitude of 82°). Two comparisons were conducted: a first one for weather stations with available temperature observations (25,576 stations covering 73,109,913 km^2^, roughly 22% of the WorldClim coverage) and a second one for weather stations with available precipitation observations (47,675 stations covering 67,893,375 km^2^, roughly 20% of the WorldClim coverage).

### Validation methodologies

We tested the relationship between MERRAclim and WorldClim bioclimatic variables calculating Pearson’s correlation coefficients and fitting linear regressions. For all versions of the 19 bioclimatic variables we found linear correlations between both datasets that explained most of the variance. Linear regressions for the 1980s and the 1990s revealed that both decades have a similar relationship with WorldClim (Supplementary File 2). The absence of WorldClim data for the 2000s prevented a comparison for this decade.

### Temperature-related bioclimatic variables (BIO1-BIO7, BIO10-BIO11)

Pearson coefficients testing the correlation between temperature-related bioclimatic variables (BIO1-BIO7, BIO10-BIO11) from WorldClim and MERRAclim were very high (>0.8) in all cases, except for BIO2 (r=0.6). BIO2, a variable representing diurnal range and thus highly sensitive to temperature extremes, showed the highest discrepancy between datasets. Overall, MERRAclim yielded higher temperature values than WorldClim with a positive and close to unity slope (Supplementary File 1). Mean temperatures of the most extreme months (BIO5 and BIO6) show the largest differences between datasets: the warmest month in MERRAclim is ~10 °C higher than in WorldClim, whereas the coldest month is around 7 °C lower. Due to this important difference between datasets we fitted linear regressions using subsets depending on the absolute difference whose geographical distribution is depicted in Supplementary Fig. 2. Firstly, we used the points that showed an absolute difference smaller than 10 °C and we obtained for BIO5 the same trend but only 7 °C higher, for BIO6 the difference was of 3 °C. Secondly, we used a subset of those points with an absolute difference smaller than 5 °C, this time the intercept for BIO5 was 5 °C and for BIO6 there were no differences with the previous subset.

### Water-related bioclimatic variables (BIO12-BIO17)

Comparisons of water-related MERRAclim bioclimatic variables for each decade with WorldClim are consistent, but important differences between versions were detected (Supplementary File 1). Bioclimatic variables from the V_min_ version show the strongest correlation with the bioclimatic variables in WorldClim and also the highest proportion of explained variance. In general, water-related bioclimatic variables were less strongly correlated with WorldClim than temperature-related ones. Bioclimatic variables describing the extreme lack of environmental water availability, both monthly (BIO14) and quarterly (BIO17), had the lowest congruence with WorldClim (Pearson correlation coefficient ~0.37 and ~0.4, respectively). Water seasonality (as described by BIO15) greatly varies in its correlation with WorldClim depending on the version, being the correlation with V_min_ four times stronger than with V_max_.

### Combined bioclimatic variables (BIO8-BIO9, BIO18-BIO19)

Combined bioclimatic variables depend on temperature and humidity information (Table 1) to describe the most extreme quarters. Although linear associations remained similar between MERRAclim versions (V_min_, V_mean_ and V_max_) and WorldClim, the strongest correlation coefficient was found for V_min_. Among temperature-dependent combined variables, BIO8 showed the greatest difference between datasets (~15 °C higher in MERRAclim). BIO9 is ~2 °C warmer in WorldClim. Water-dependent combined variables (BIO18 and BIO19) followed the same trends as other water-related variables and, again, the V_min_ version showed the highest variance explained (Supplementary File 1).

### Geographic location of the differences between WorldClim and MERRAclim

We located those geographical areas where MERRAclim (V_min_ version) and WorldClim vary the most using the outliers of residuals from linear regressions for each bioclimatic variable (Supplementary Figs 3 and 4). We defined outliers using the IQR (InterQuartile Range) of the residuals, for which we calculated the first and third quartiles (Q1 and Q3) and estimated the values outside the range Q1—(1.5*IQR) and Q3+(1.5*IQR) as outliers. Both datasets showed an outstanding spatial congruence and the average area of outliers for each bioclimatic variable covers less than 5% of the compared geographical space (only BIO9 has a larger extent of outliers, summing up to 7%, Supplementary File 1). To get a more detailed information of these variations we have also drawn the bias from the fitted linear regressions (Supplementary Fig. 5).

The differences between the datasets, as identified by the outliers and the bias, are geographically clustered (Supplementary Figs 3 and 4); which can probably be explained by the fact that WorldClim was built from heterogeneous regional networks of weather stations some of which are also compiled from several datasets^48^ (e.g., Latin America, The Caribbean, the Altiplano in Peru and Bolivia, European Nordic Countries, the United States of America, Australia, New Zealand and Madagascar) that depend on different sources of information and techniques^16^.

## Additional Information

**How to cite this article:** Vega, G. C. *et al.* MERRAclim, a high-resolution global dataset of remotely sensed bioclimatic variables for ecological modelling. *Sci. Data* 4:170078 doi: 10.1038/sdata.2017.78 (2017).

**Publisher’s note:** Springer Nature remains neutral with regard to jurisdictional claims in published maps and institutional affiliations.

## Supplementary Material

Supplementary Information

Supplementary Information

## Figures and Tables

**Table 1:** Summary of MERRAclim’s bioclimatic variables.

**Variable shortname**	**Variable description**	**Units**	**Variable type**	**Resolution**	**Version**	**Decade**	**Naming convention**	**Example**
BIO1	Annual Mean Temperature	*10 °C	temperature-related	10 m, 5 m, 2.5 m	min, mean, max	80 s, 90 s, 00 s	resolution_version_decade_BIO1.tif	10m_min_00s_BIO1.tif (BIO1 from 00 s decade at 10 m resolution from version Vmin)
BIO2	Mean Diurnal Range Temperature	*10 °C	temperature-related	10 m, 5 m, 2.5 m	min, mean, max	80 s, 90 s, 00 s	resolution_version_decade_BIO2.tif	2_5m_mean_90s_BIO2.tif (BIO2 from 90 s decade at 2.5 m resolution from version Vmean)
BIO3	Isothermality (BIO2/BIO7) (* 100)	*10 °C	temperature-related	10 m, 5 m, 2.5 m	min, mean, max	80 s, 90 s, 00 s	resolution_version_decade_BIO3.tif	10m_max_80s_BIO3.tif (BIO3 from 80 s decade at 10 m resolution from version Vmax)
BIO4	Temperature Seasonality(standard deviation *100)	*10 °C	temperature-related	10 m, 5 m, 2.5 m	min, mean, max	80 s, 90 s, 00 s	resolution_version_decade_BIO4.tif	5m_min_00s_BIO4.tif (BIO4 from 00 s decade at 5 m resolution from version Vmin)
BIO5	Max Temperature of Warmest Month	*10 °C	temperature-related	10 m, 5 m, 2.5 m	min, mean, max	80 s, 90 s, 00 s	resolution_version_decade_BIO5.tif	2_5_mean_90s_BIO5.tif (BIO5 from 90 s decade at 2.5 m resolution from version Vmean)
BIO6	Min Temperature of Coldest Month	*10 °C	temperature-related	10 m, 5 m, 2.5 m	min, mean, max	80 s, 90 s, 00 s	resolution_version_decade_BIO6.tif	10m_min_80s_BIO6.tif (BIO6 from 80 s decade at 10 m resolution from version Vmin)
BIO7	Temperature Annual Range (BIO5-BIO6)	*10 °C	temperature-related	10 m, 5 m, 2.5 m	min, mean, max	80 s, 90 s, 00 s	resolution_version_decade_BIO7.tif	5m_max_00s_BIO7.tif (BIO7 from 00 s decade at 5 m resolution from version Vmax)
BIO8	Mean temperature of most humid quarter	*10 °C	combined	10 m, 5 m, 2.5 m	min, mean, max	80 s, 90 s, 00 s	resolution_version_decade_BIO8.tif	10m_mean_90s_BIO8.tif (BIO8 from 90 s decade at 10 m resolution from version Vmean)
BIO9	Mean temperature of least humid quarter	*10 °C	combined	10 m, 5 m, 2.5 m	min, mean, max	80 s, 90 s, 00 s	resolution_version_decade_BIO9.tif	2_5m_min_80s_BIO9.tif (BIO9 from 80 s decade at 2.5 m resolution from version Vmin)
BIO10	Mean Temperature of Warmest Quarter	*10 °C	temperature-related	10 m, 5 m, 2.5 m	min, mean, max	80 s, 90 s, 00 s	resolution_version_decade_BIO10.tif	5m_max_00s_BIO10.tif (BIO10 from 00 s decade at 5 m resolution from version Vmax)
BIO11	Mean Temperature of Coldest Quarter	*10 °C	temperature-related	10 m, 5 m, 2.5 m	min, mean, max	80 s, 90 s, 00 s	resolution_version_decade_BIO11.tif	2_5m_mean_80s_BIO11.tif (BIO11 from 80 s decade at 2.5 m resolution from version Vmean)
BIO12	Annual Mean Specific Humidity	100000 * kg of water/kg of air	water-related	10 m, 5 m, 2.5 m	min, mean, max	80 s, 90 s, 00 s	resolution_version_decade_BIO12.tif	10m_min_90s_BIO12.tif (BIO12 from 90 s decade at 10 m resolution from version Vmin)
BIO13	Specific Humidity of most humid Month	100000 * kg of water/kg of air	water-related	10 m, 5 m, 2.5 m	min, mean, max	80 s, 90 s, 00 s	resolution_version_decade_BIO13.tif	5m_max_80s_BIO13.tif (BIO13 from 80 s decade at 5 m resolution from version Vmax)
BIO14	Specific Humidity of least humid Month	100000 * kg of water/kg of air	water-related	10 m, 5 m, 2.5 m	min, mean, max	80 s, 90 s, 00 s	resolution_version_decade_BIO14.tif	5m_mean_90s_BIO14.tif (BIO14 from 90 s decade at 5 m resolution from version Vmean)
BIO15	Specific Humidity seasonality (Coefficient of variation)	100000 * kg of water/kg of air	water-related	10 m, 5 m, 2.5 m	min, mean, max	80 s, 90 s, 00 s	resolution_version_decade_BIO15.tif	2_5m_min_00s_BIO15.tif (BIO15 from 00 s decade at 2.5 m resolution from version Vmin)
BIO16	Specific Humidity Mean of most humid quarter	100000 * kg of water/kg of air	water-related	10 m, 5 m, 2.5 m	min, mean, max	80 s, 90 s, 00 s	resolution_version_decade_BIO16.tif	5m_mean_90s_BIO16.tif (BIO16 from 90 s decade at 5 m resolution from version Vmean)
BIO17	Specific Humidity Mean of least humid quarter	100000 * kg of water/kg of air	water-related	10 m, 5 m, 2.5 m	min, mean, max	80 s, 90 s, 00 s	resolution_version_decade_BIO17.tif	10m_max_80s_BIO17.tif (BIO17 from 80 s decade at 10 m resolution from version Vmax)
BIO18	Specific Humidity Mean of warmest quarter	100000 * kg of water/kg of air	combined	10 m, 5 m, 2.5 m	min, mean, max	80 s, 90 s, 00 s	resolution_version_decade_BIO18.tif	5m_mean_00s_BIO18.tif (BIO18 from 00 s decade at 5m resolution from version Vmean)
BIO19	Specific Humidity Mean of coldest quarter	100000 * kg of water/kg of air	combined	10 m, 5 m, 2.5 m	min, mean, max	80 s, 90 s, 00 s	resolution_version_decade_BIO19.tif	2_5m_max_00s_BIO19.tif (BIO19 from 00 s decade at 2.5 m resolution from version Vmax)
There are 11 temperature-related variables of which two are combined and 7 water-related variables of which two are combined.								

**Table 2:** Summary of the references evaluating and comparing the water and energy variables of the MERRA dataset.

**Reference**	**Covered Dates**	**Variables**	**Location**
Ashouri *et al.*^33^	1979–2010	Water	USA
Bracegirdle & Marshall^43^	1979–2008	Energy	Antarctica
Essou *et al.*^34^	1979–2003	Water and Energy	USA
Lader *et al.*^45^	1979–2009	Water and Energy	Alaska (USA)
Bosilovich *et al.*^37^	1979–2012	Water	Central USA
Roberts *et al.*^35^	2000–2010	Water	West Africa
Lindsay *et al.*^42^	1981–2010	Water and Energy	Arctic
Lorenz *et al.*^36^	1989–2010	Water	Global
Bosilovich *et al.*^39^	1979–2009	Water and Energy	Global
Trenberth *et al.*^38^	1979–2005	Water	Global
Cullather & Bosilovich^40^	1979–2005	Water	Polar Regions
Cullather & Bosilovich^44^	1979–2005	Energy	Polar Regions
Serreze *et al.*^41^	1979–2010	Water and Energy	Arctic

**Table 3:** Description of the time series from the United States Antarctic Program meteorological stations used to fit linear regressions and to calculate Pearson’s correlation.

**Location**	**Region**	**Time span**	**Number of records**	**Time step**	**Pearson’s correlation**	**Slope**	**Intercept**	**Explained variance**	**Bias**
Palmer	East Antarctica	2007–10	31066	1 h	0.82	0.86	−1.57 °C	0.68	±2.4 °C
McMurdo	West Antarctica	1990s	10865	6 h	0.87	0.73	−8.9 °C	0.76	±5.4 °C
Amundsen South Pole	Interior Antarctica	1990s	87618	1 h	0.92	0.69	−11 °C	0.86	±4.5 °C

**Table 4:** Temperature-only bioclimatic variables computed with MERRA data and United States Antarctic Program (USAP) meteorological stations.

**Location**	**Dataset**	**bio1**	**bio2**	**bio3**	**bio4**	**bio5**	**bio6**	**bio7**	**bio10**	**bio11**
Palmer	MERRA	−4.3	11.3	47.7	346.0	3.2	−20.6	23.8	0.0	−8.5
	USAP	−1.3	12.1	48.3	345.4	8.8	−16.1	25.0	2.9	−5.6
McMurdo	MERRA	−21.3	18.9	47.7	721.4	−1.7	−41.2	39.6	−11.4	−28.2
	USAP	−16.0	20.9	48.1	808.8	4.8	−38.7	43.5	−4.9	−23.5
Amundsen South Pole	MERRA	−45.3	19.3	45.4	907.8	−23.1	−65.9	42.8	−32.1	−53.2
	USAP	−48.5	27.7	49.9	1130.2	−21.0	−76.6	55.6	−32.2	−58.0
The unit for all the values is °C. NOTE: For Palmer station only records from 2007 to 2009 have been used.										
