# Erratum: MERRAclim, a high-resolution global dataset of remotely sensed bioclimatic variables for ecological modelling

**DOI:** 10.1038/sdata.2018.70

**Published:** 2018-04-17

**Authors:** Greta C Vega, Luis R Pertierra, Miguel Ángel Olalla-Tárraga

*Scientific Data* **4**:170078 doi: 10.1038/sdata.2017.78 (2017); Published 20 June 2017; Updated 17 April 2018.

Supplementary File 1 was accidentally excluded from this Data Descriptor. It has now been included.

## Supplementary Material

Supplementary File 1

