# Supplementary material for: MERRAclim, a high-resolution global dataset of remotely sensed bioclimatic variables for ecological modelling
Source: Sci Data. 2017 Jun 20;4:170078. doi: 10.1038/sdata.2017.78 (PMC5477563; doi:10.1038/sdata.2017.78)

Supplementary Information

Table of contents:

Supplementary Figure 1	page 2
Supplementary Figure 2	page 12
Supplementary Figure 3	page 13
Supplementary Figure 4	page 14
Supplementary Figure 5	page 15

Supplementary Figure 1: Absolute difference between MERRAclim interpolated using Kriging and MERRAclim interpolated using Spline at 10 arc-minutes resolution

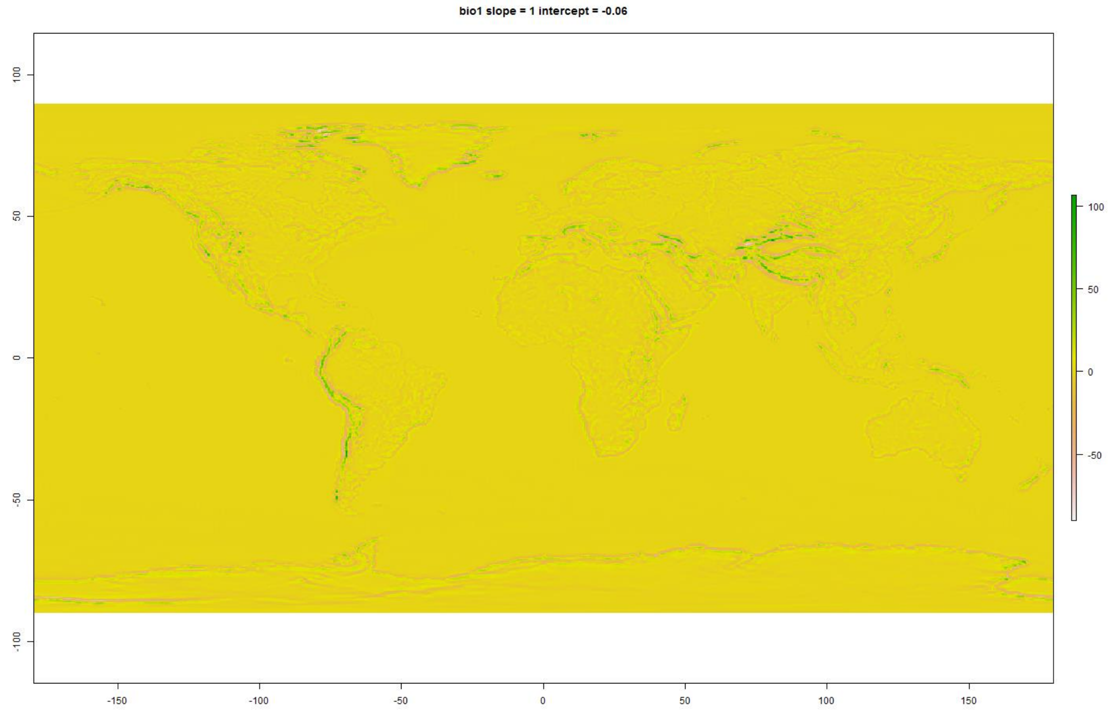

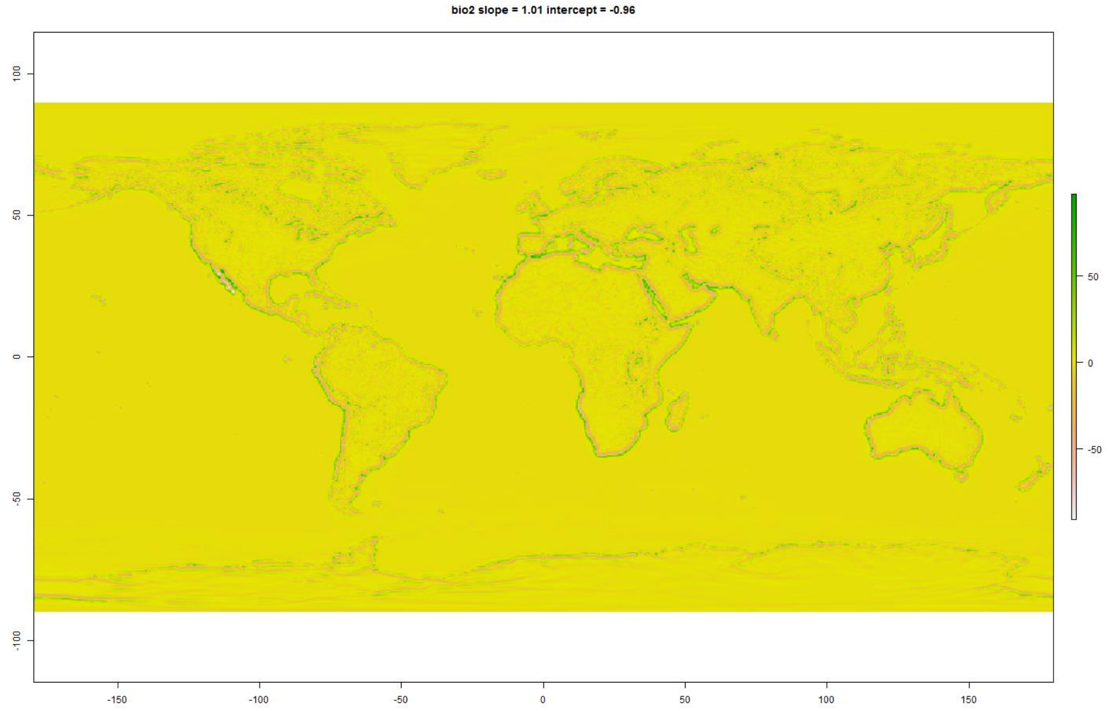

Supplementary Figure 1 (cont.)

bio3 slope = 1.02 intercept = -0.7

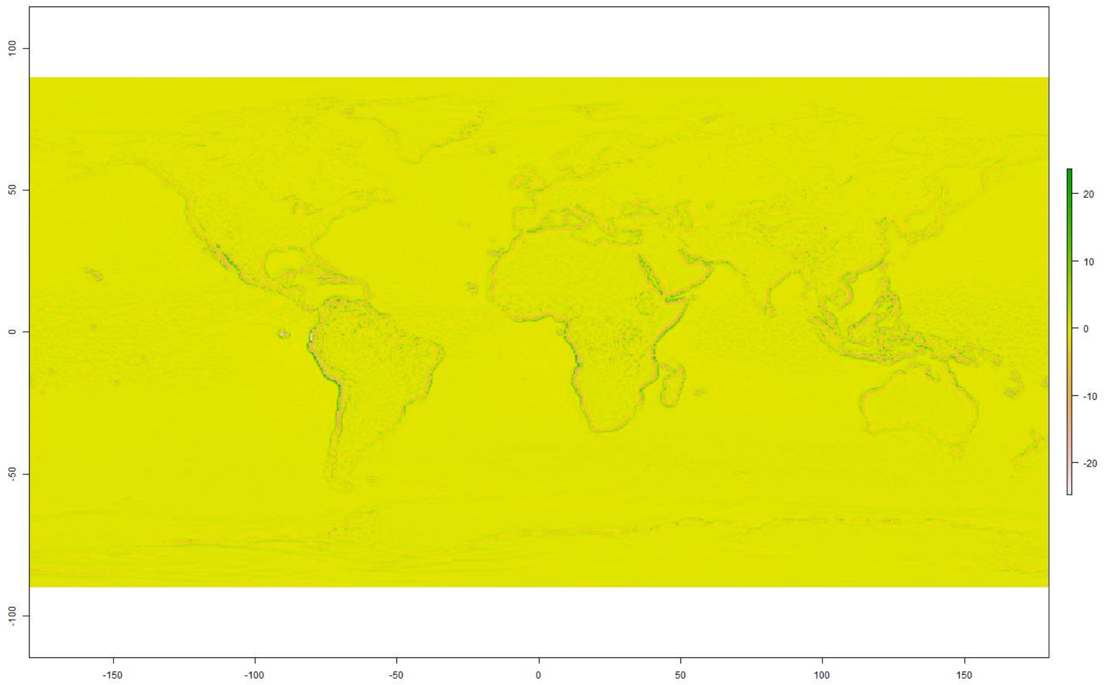

bio4 slope = 1 intercept = -12.57

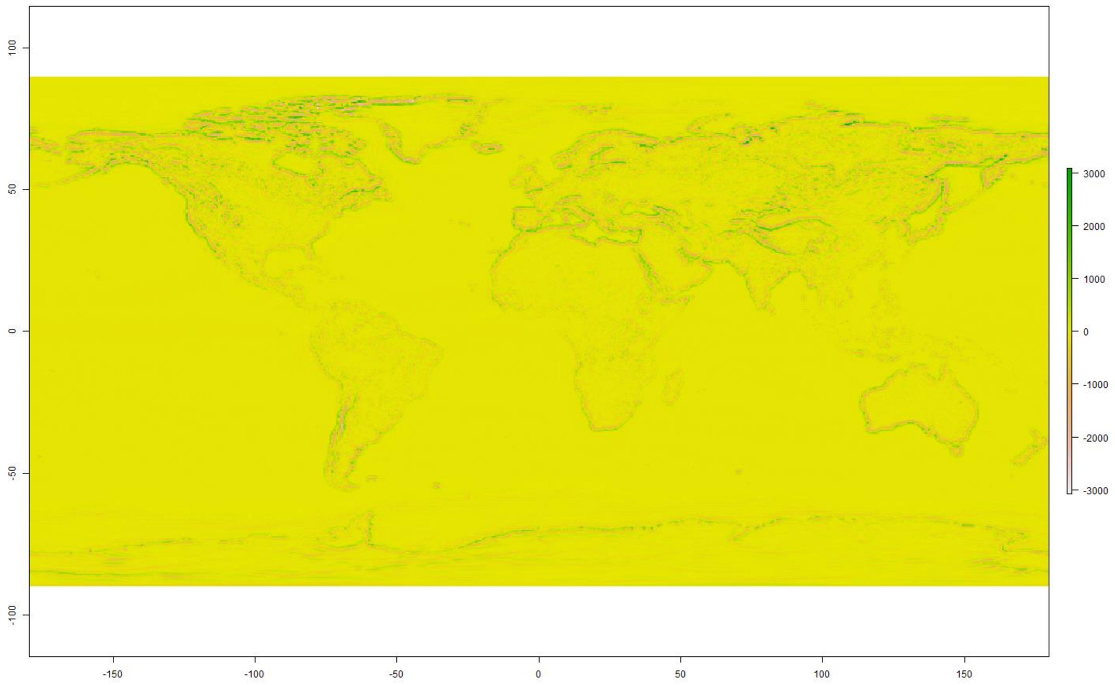

Supplementary Figure 1 (cont.)

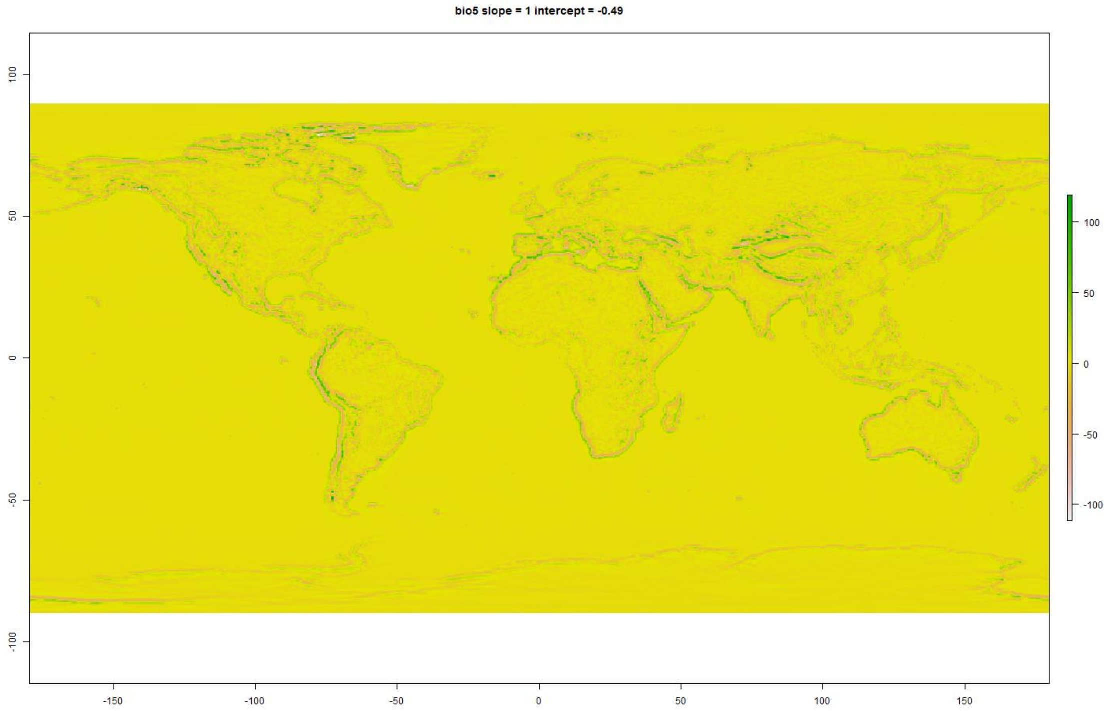

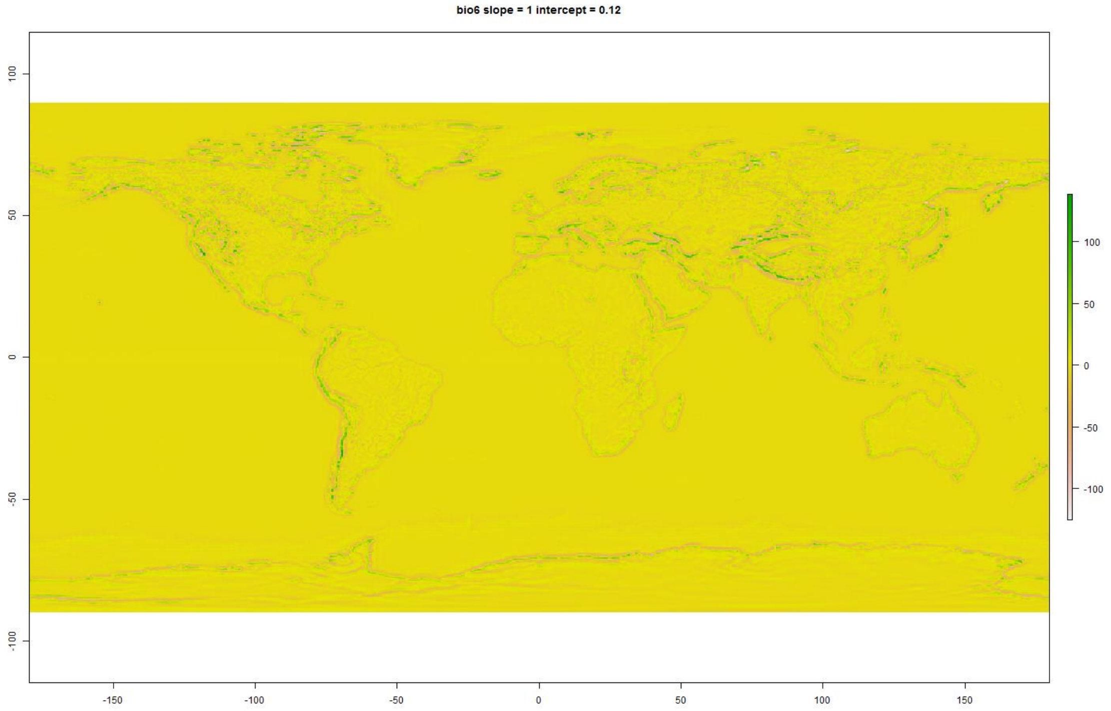

Supplementary Figure 1 (cont.)

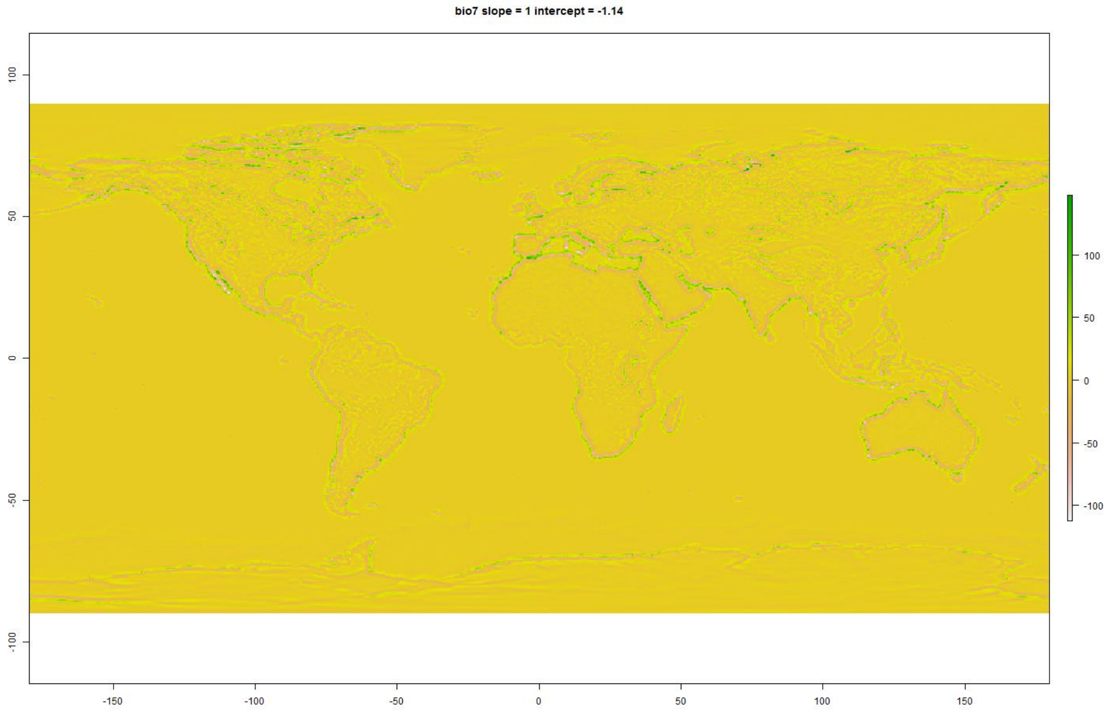

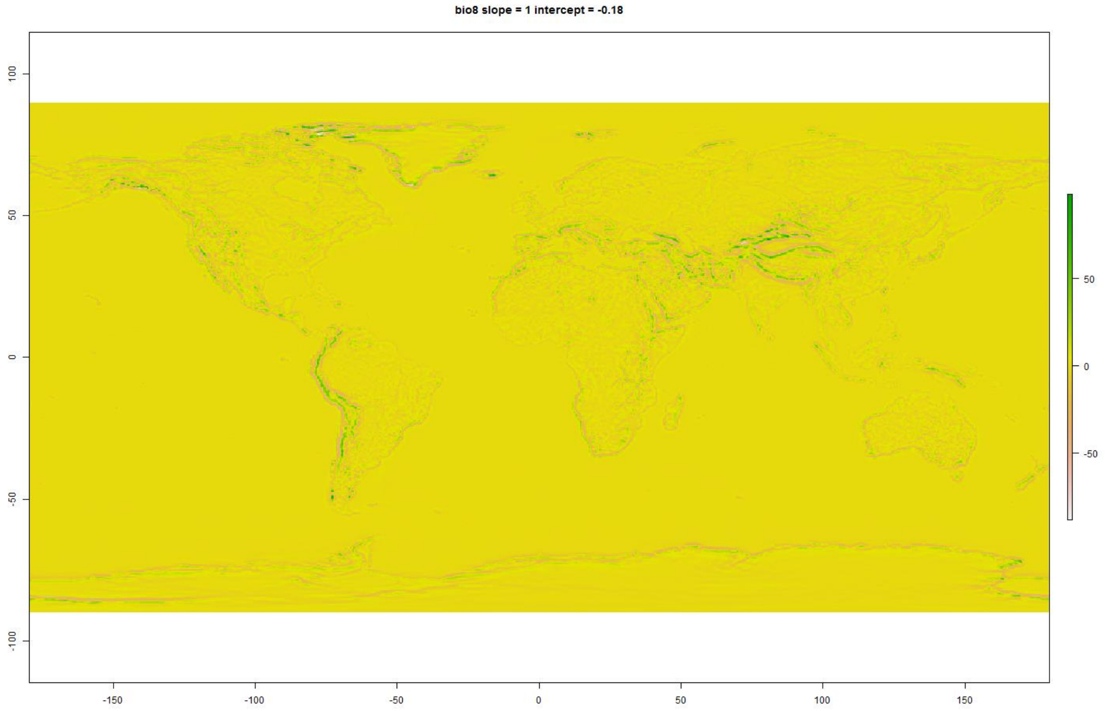

Supplementary Figure 1 (cont.)

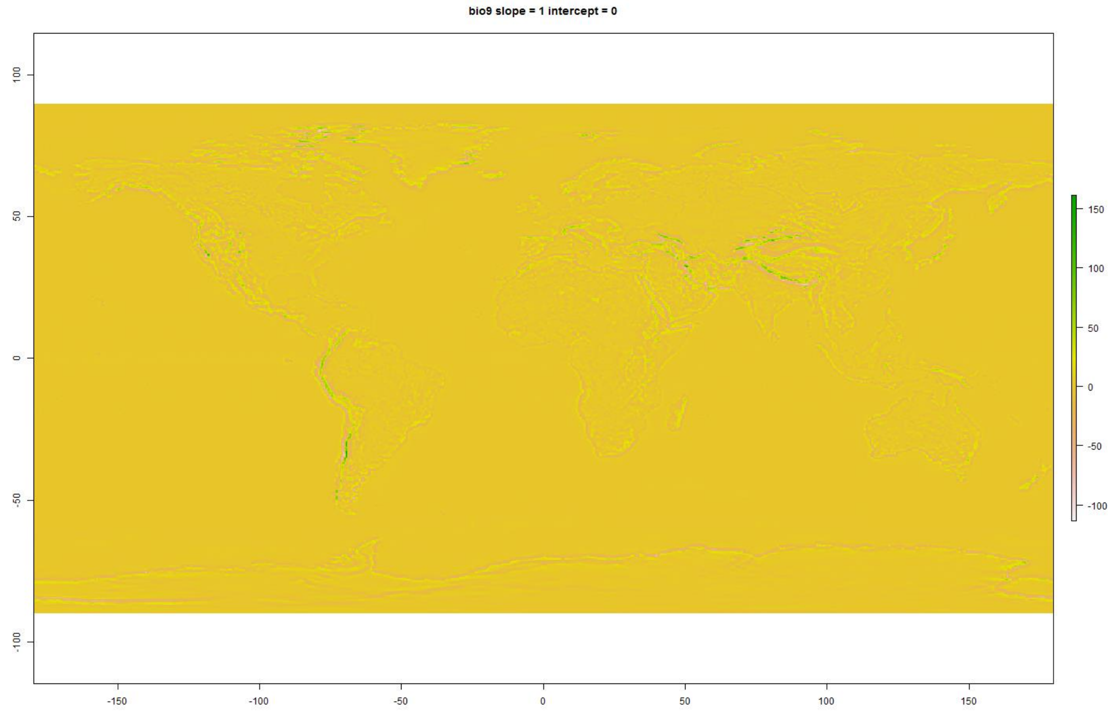

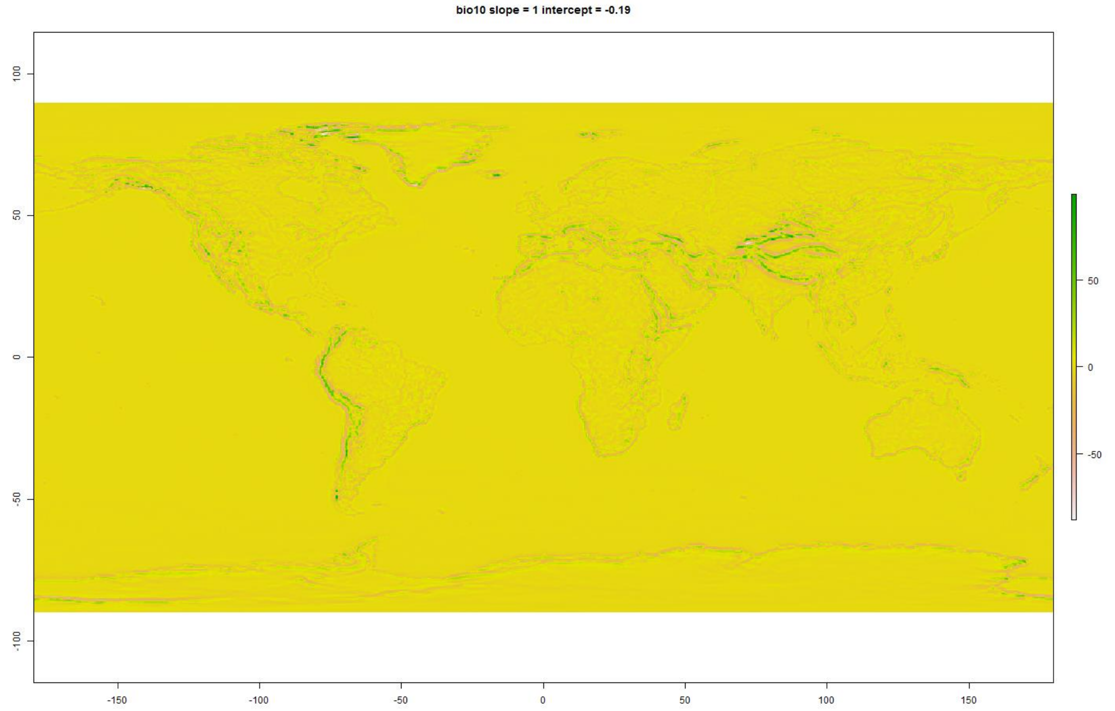

Supplementary Figure 1 (cont.)

bio11 slope = 1 intercept = 0.01

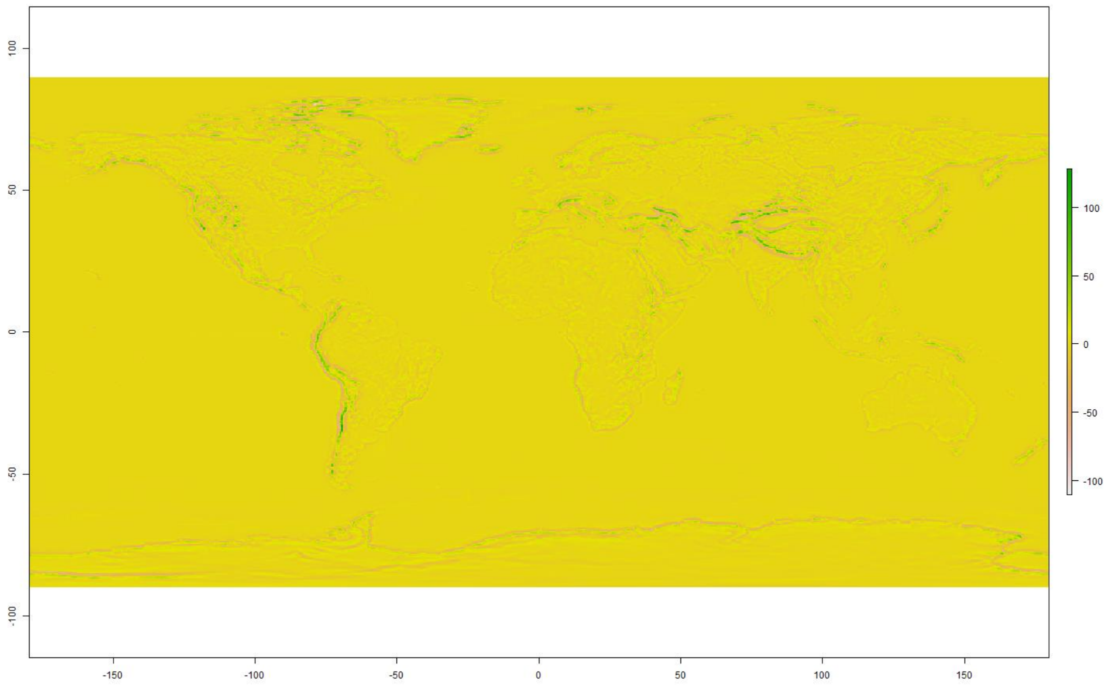

bio12 slope = 0.08 intercept = 0

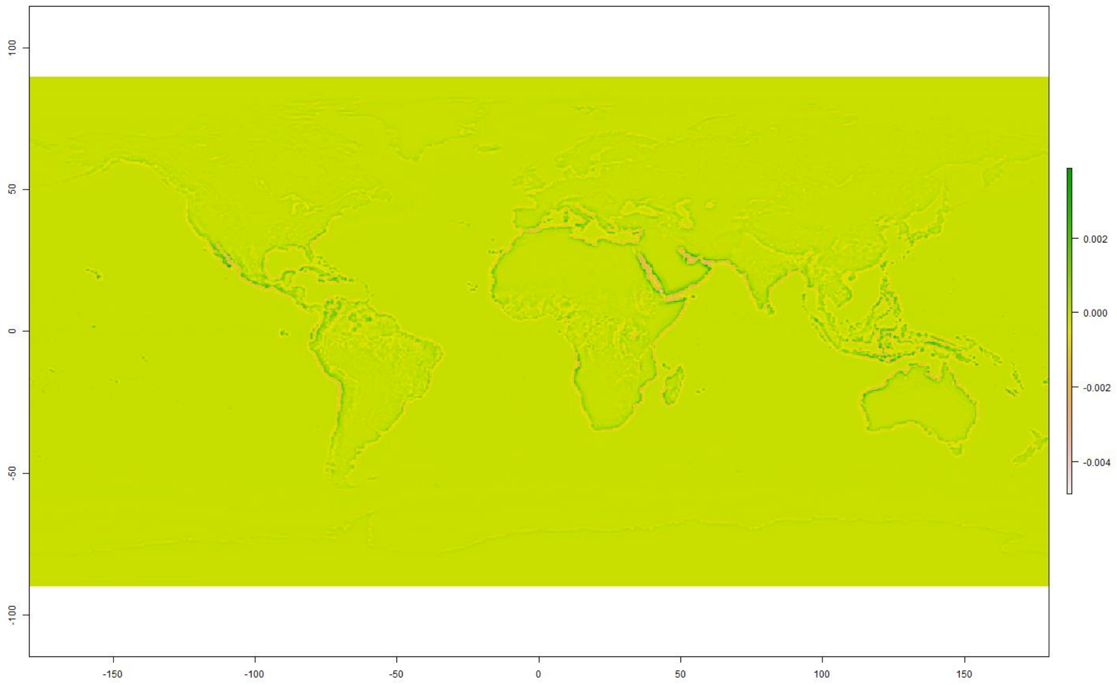

Supplementary Figure 1 (cont.)

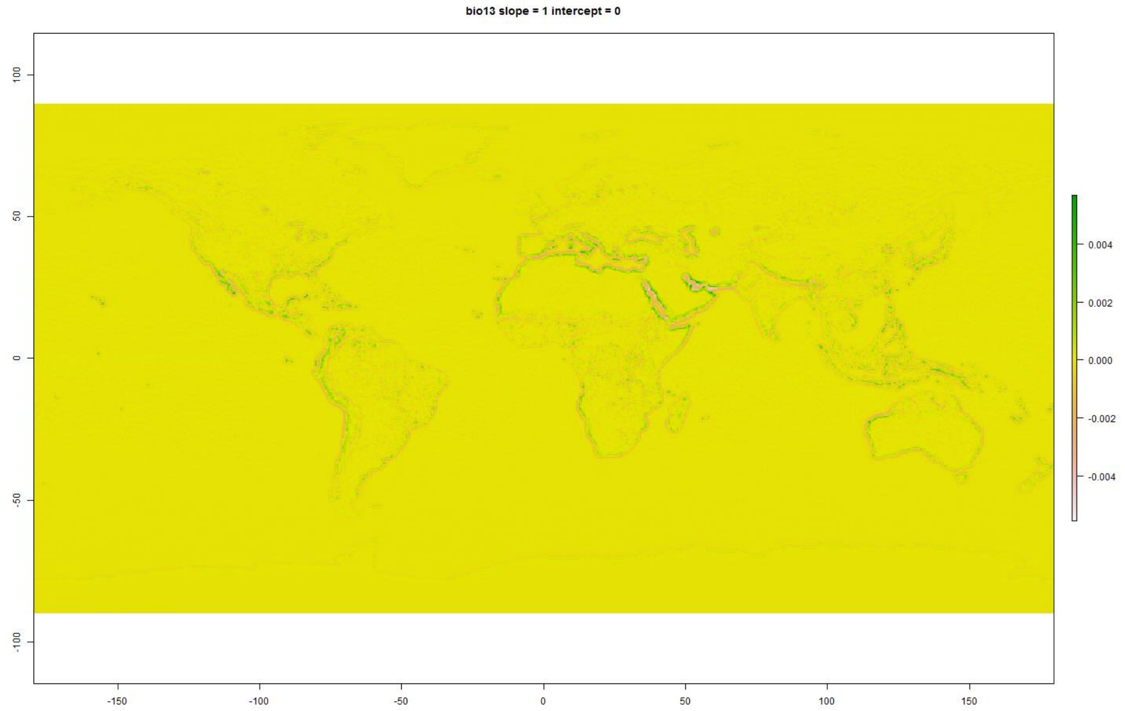

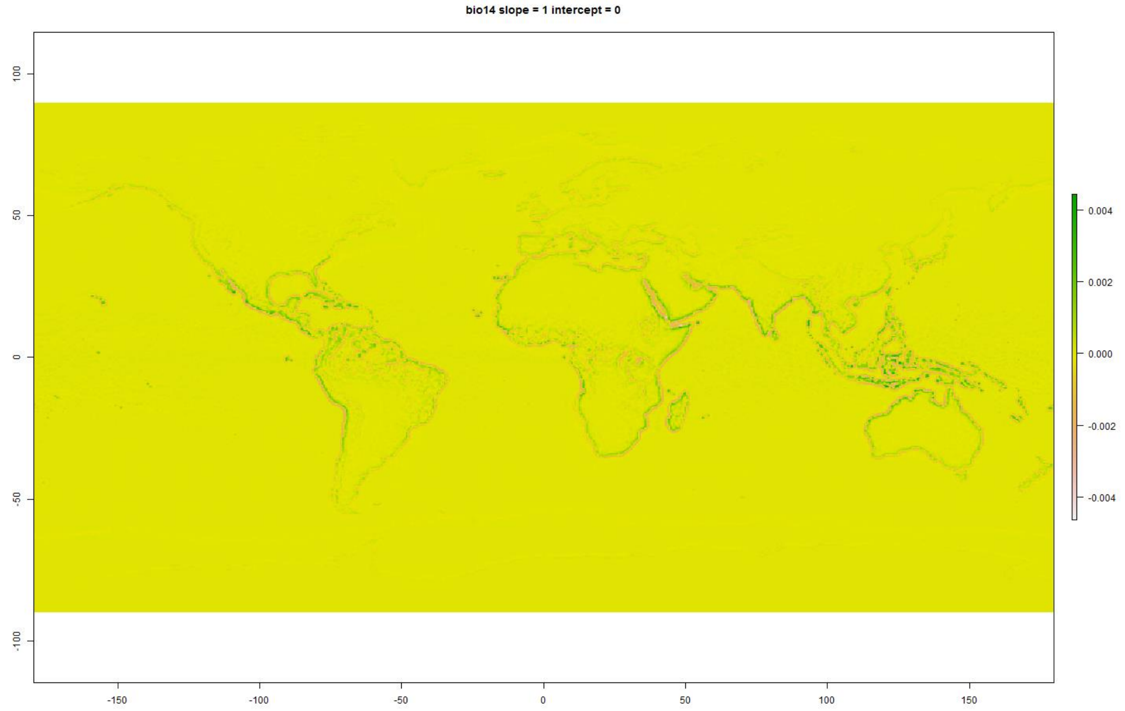

Supplementary Figure 1 (cont.)

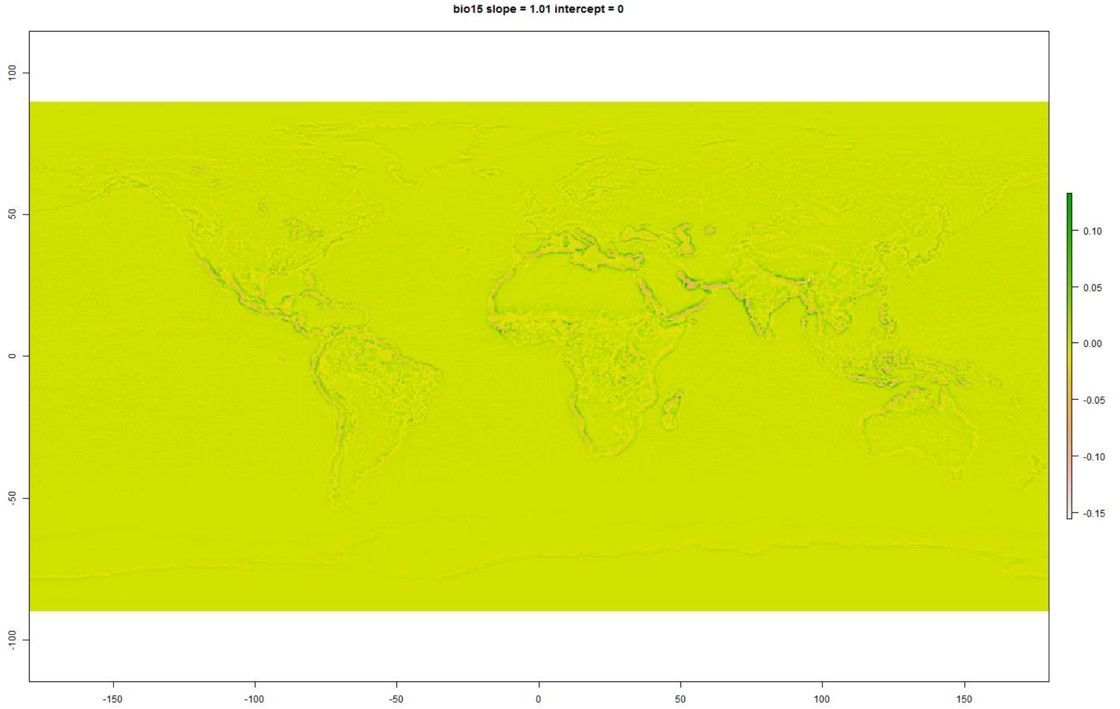

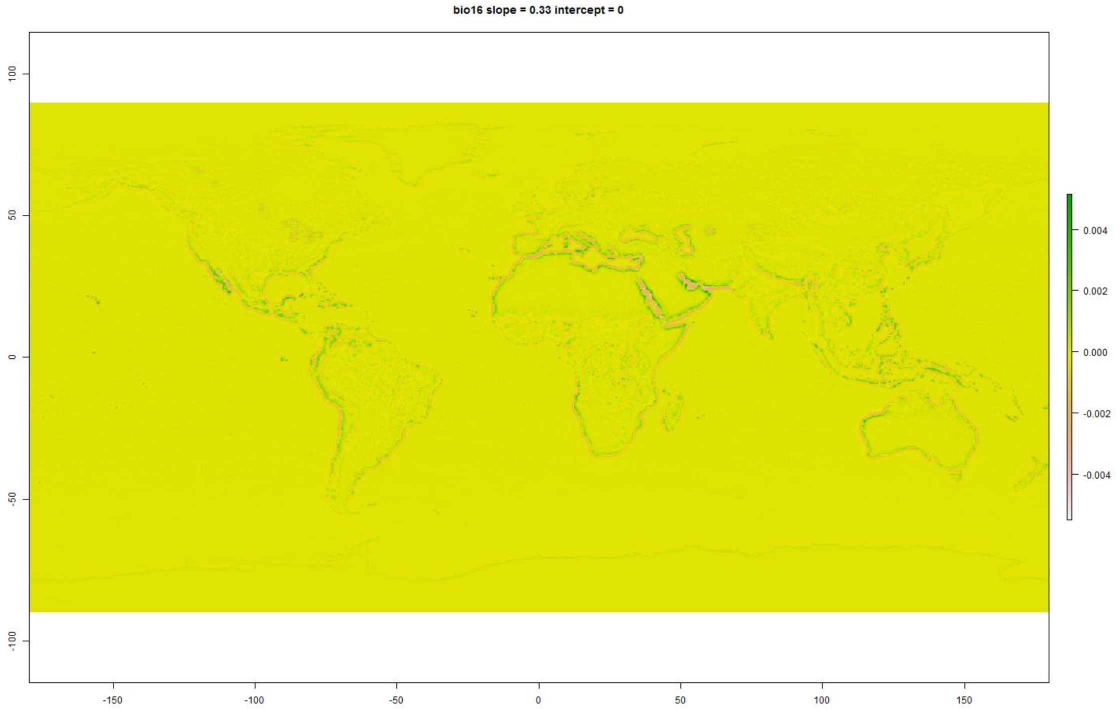

Supplementary Figure 1 (cont.)

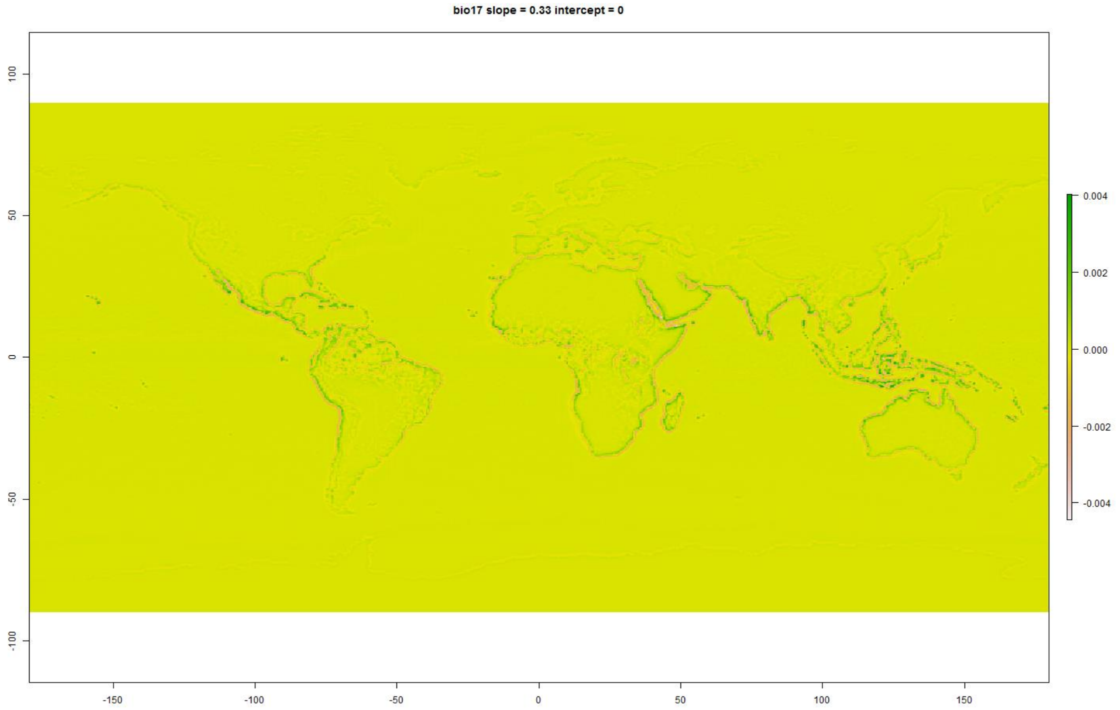

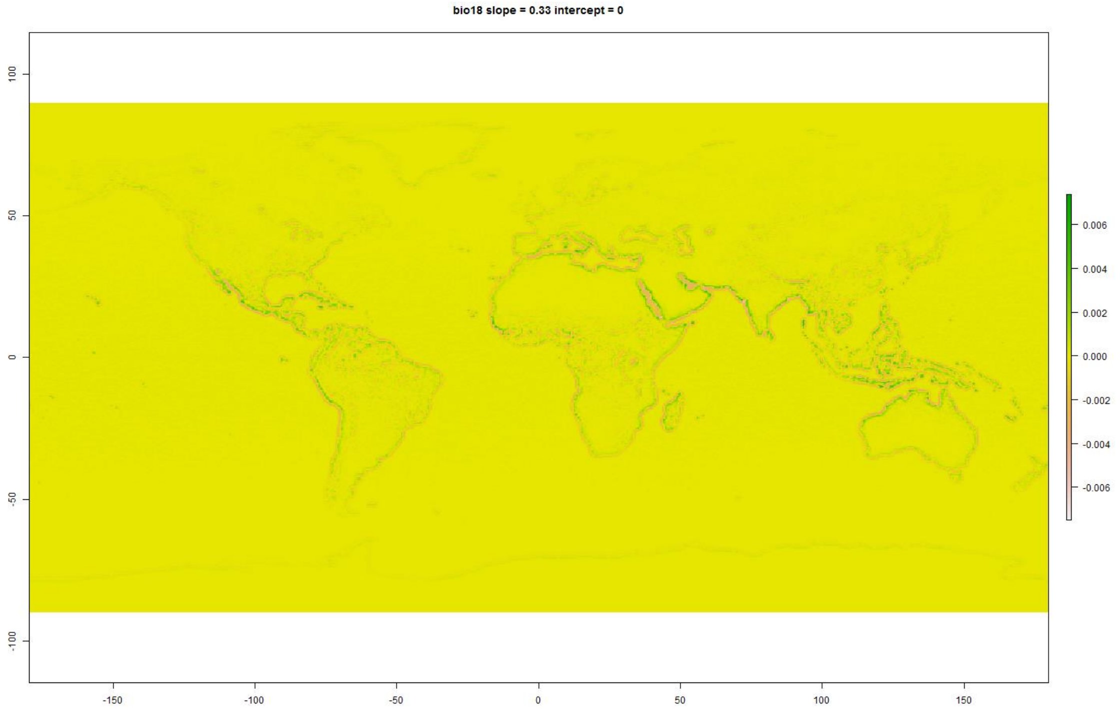

Supplementary Figure 1 (cont.)

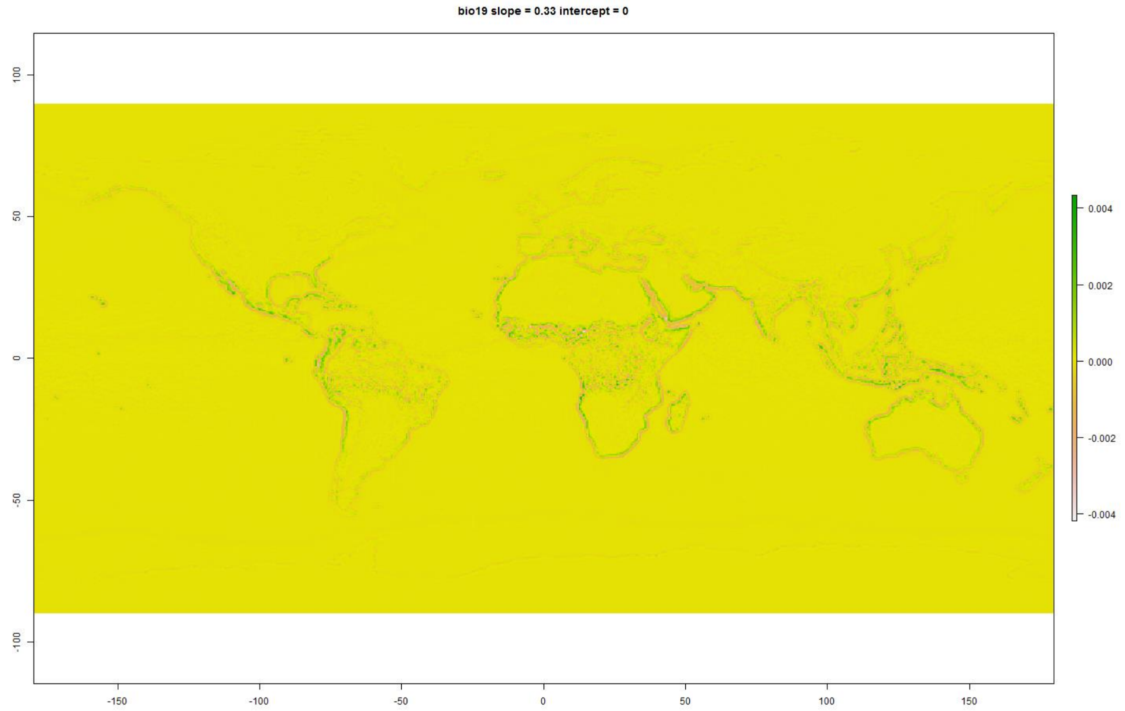

Supplementary Figure 2: Geographical location of absolute difference between MERRAclim and WorldClim for BIO5 and BIO6.

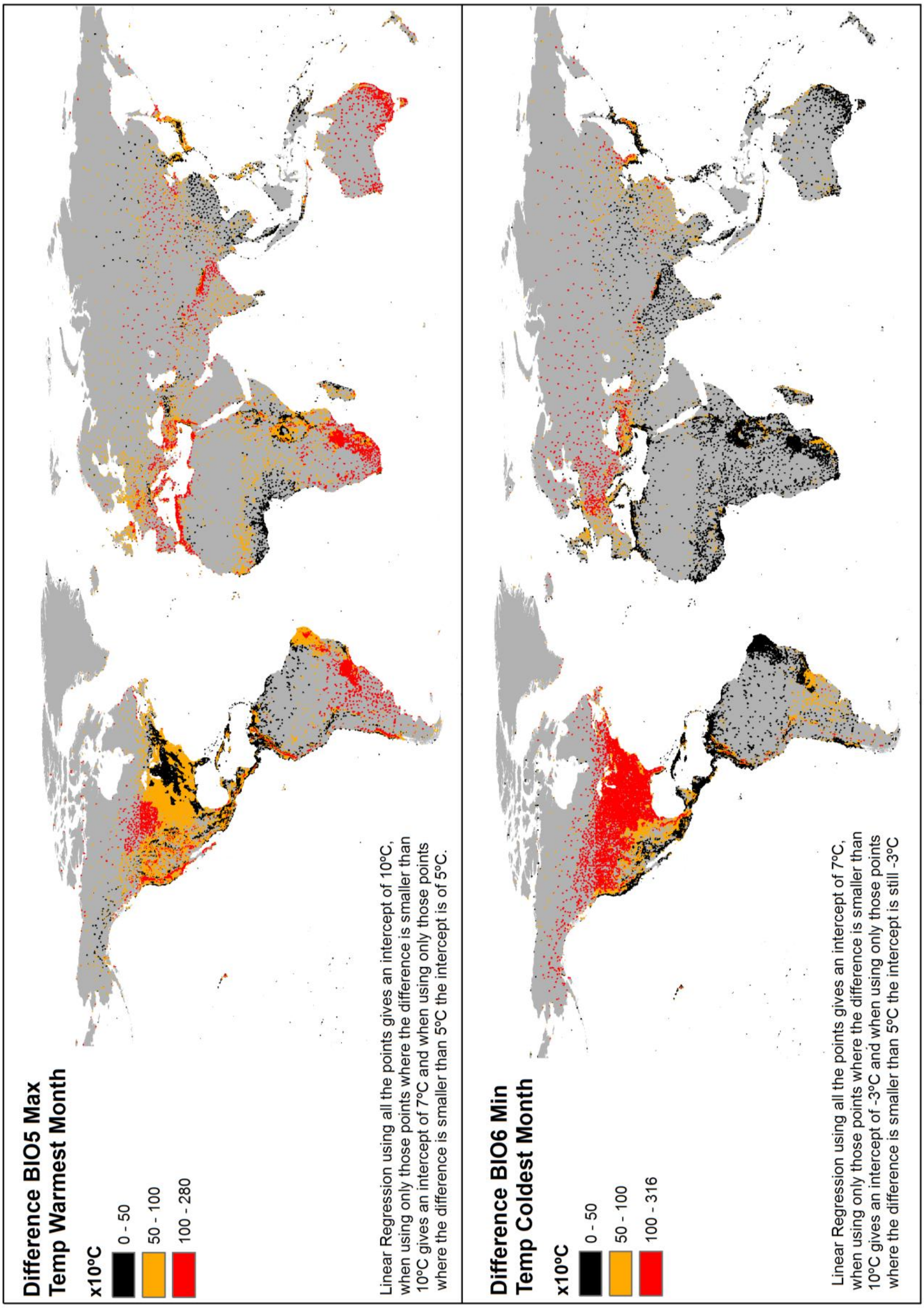

Supplementary Figure 3: Geographical location of residual outliers (red areas) from the comparison of MERRAclim vs WorldClim for each temperature – related bioclimatic variable. Black areas represent the area of comparison.

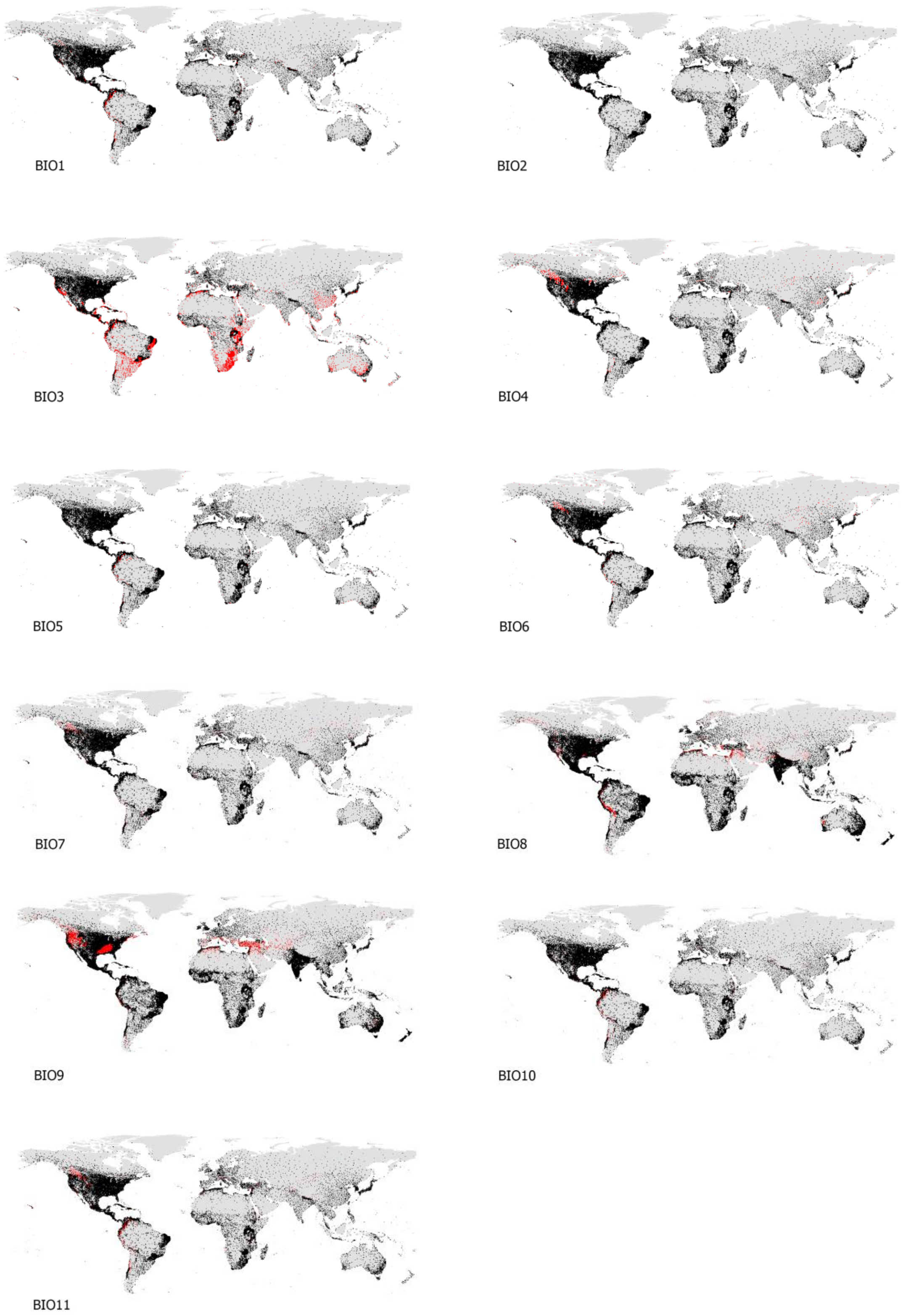

Supplementary Figure 4: Geographical location of residual outliers (red areas) from the comparison of MERRAclim vs WorldClim for each water – related bioclimatic variable. Black areas represent the area of comparison.

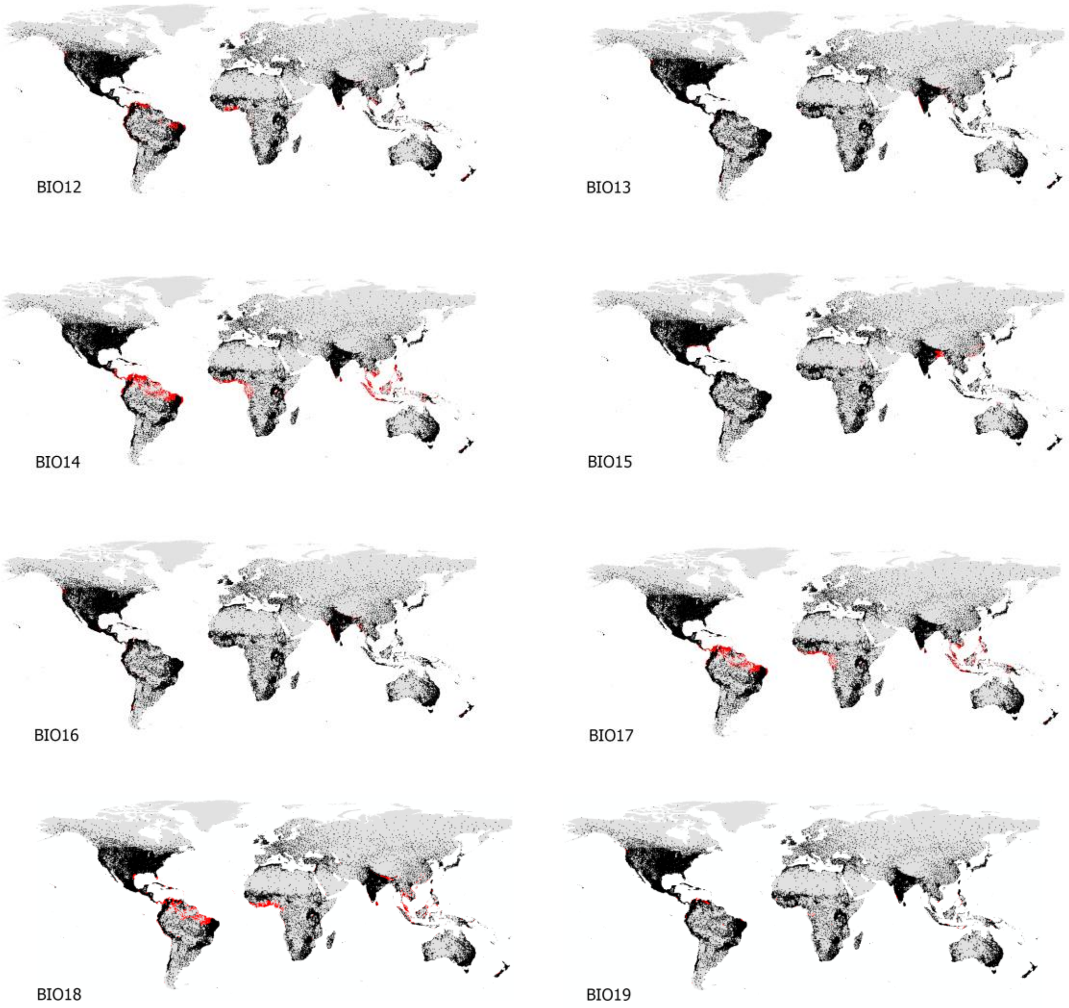

Supplementary Figure 5: Geographical location of bias for linear regressions from the comparison of MERRAclim vs WorldClim for all the only-temperature bioclimatic variables.

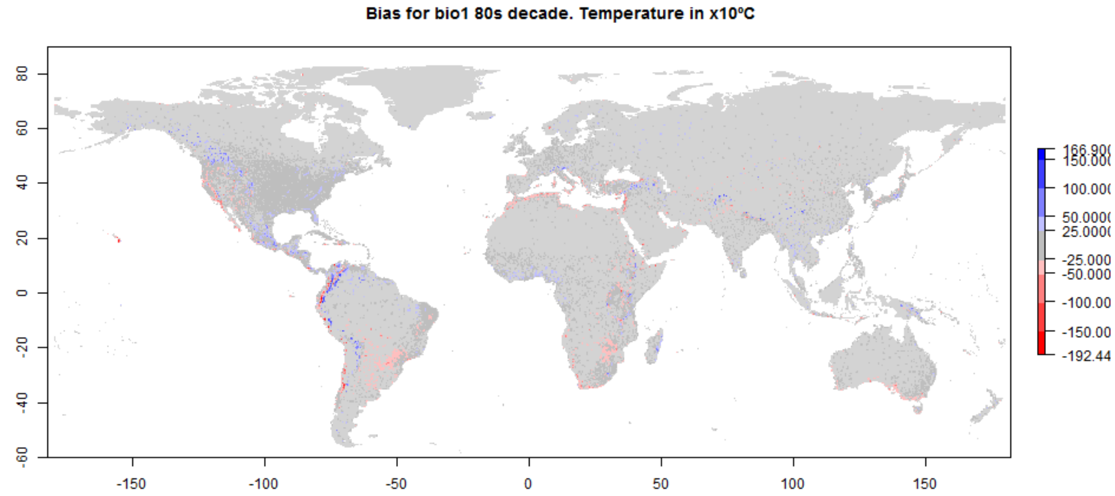

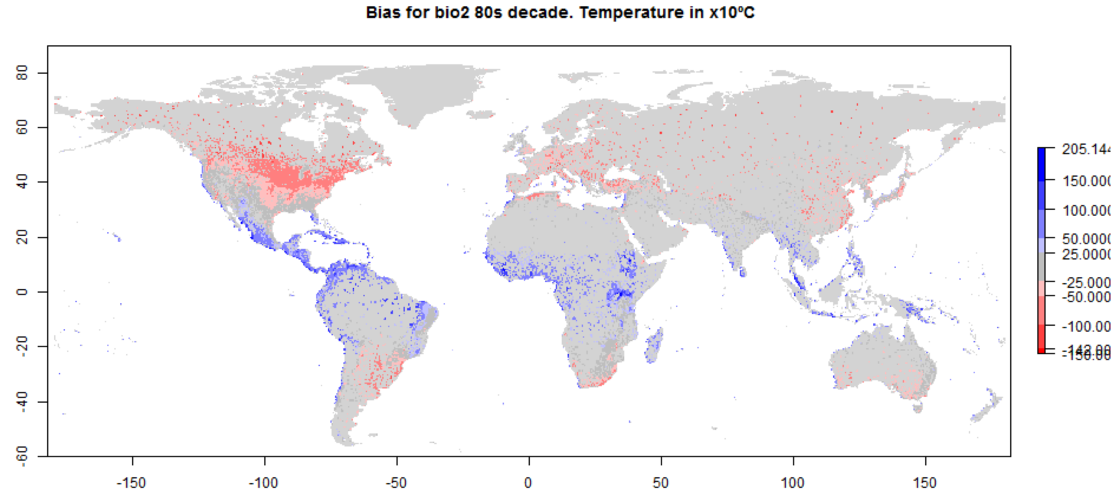

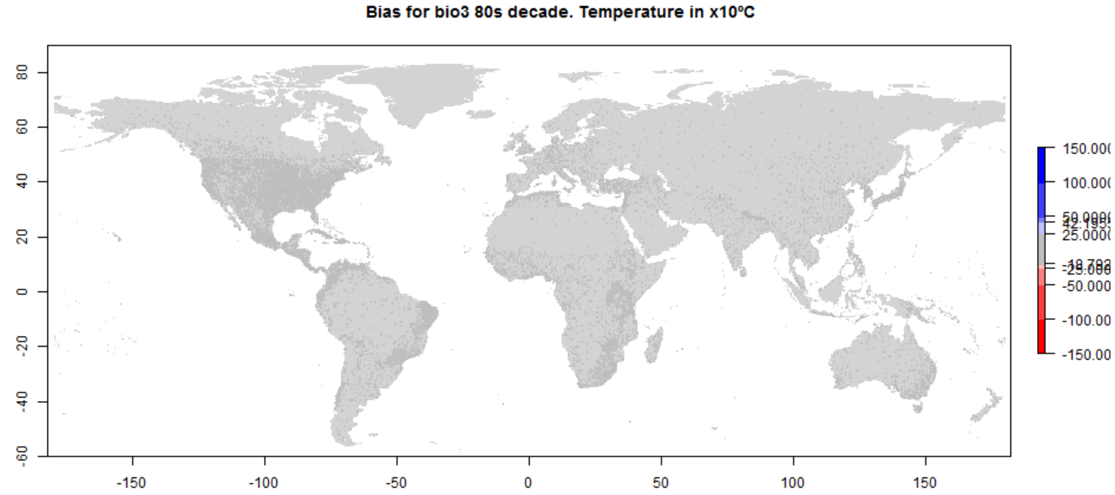

Supplementary Figure 5 (cont.)

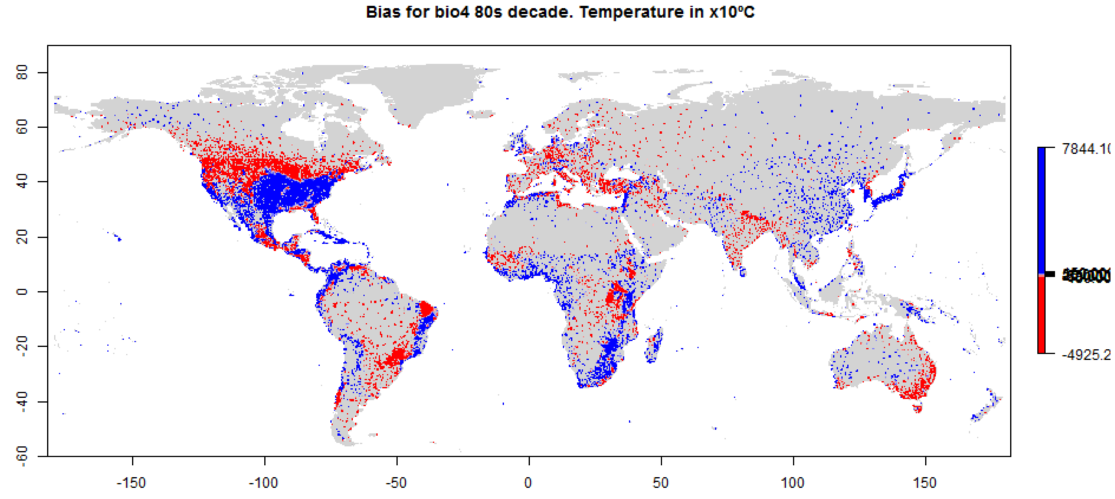

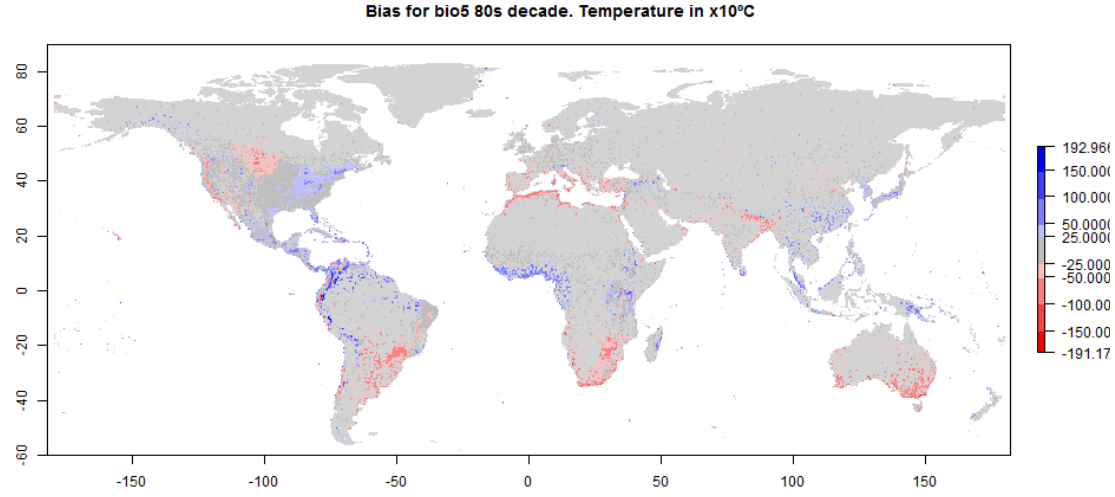

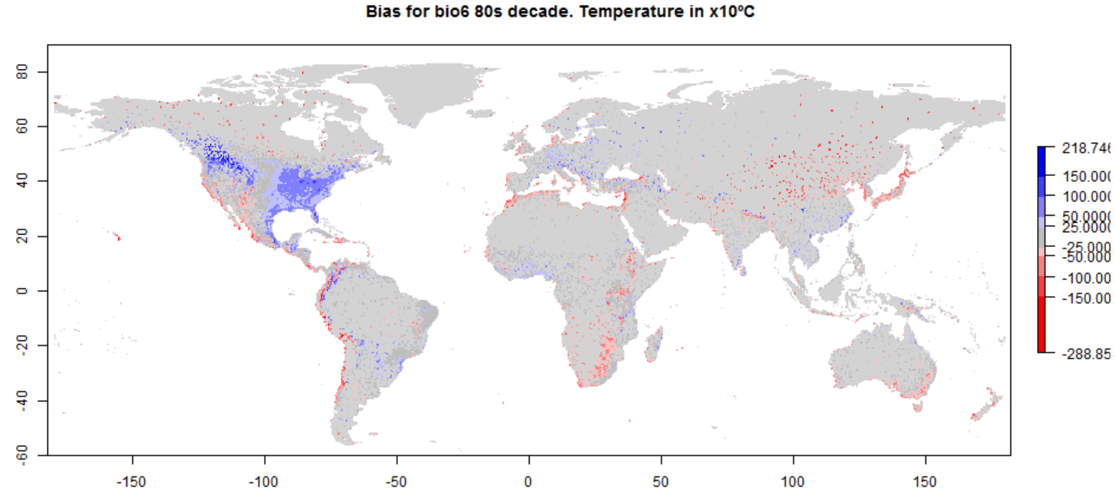

Supplementary Figure 5 (cont.)

Bias for bio7 80s decade. Temperature in x10ºC

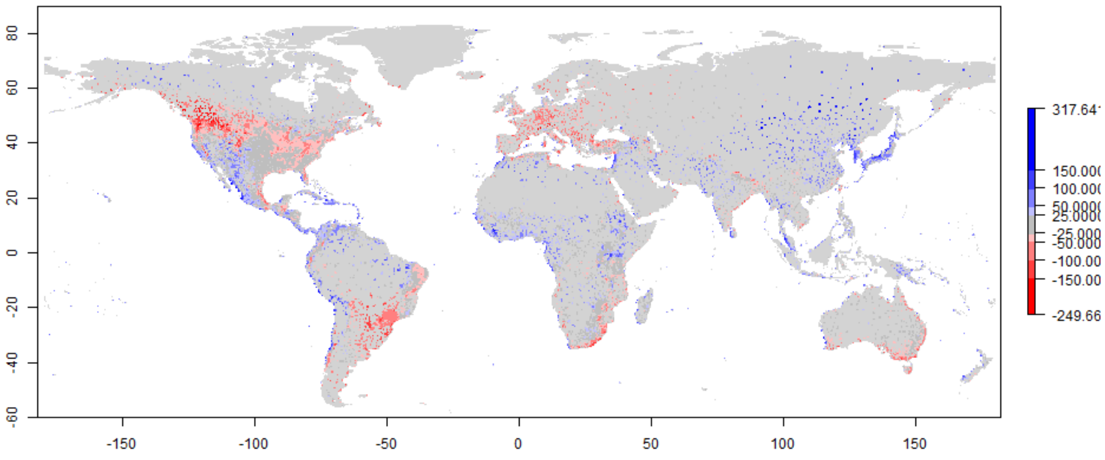

Bias for bio10 80s decade. Temperature in x10ºC

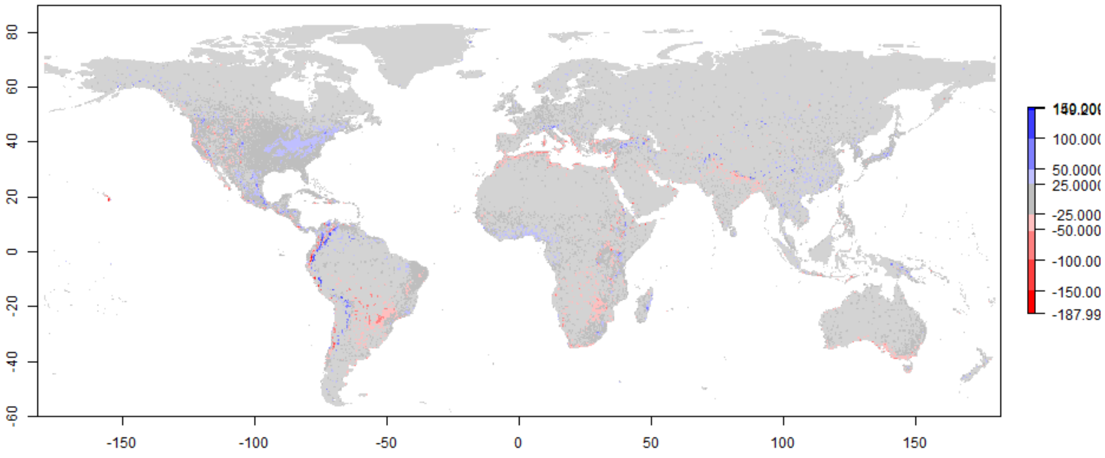

Bias for bio11 80s decade. Temperature in x10ºC

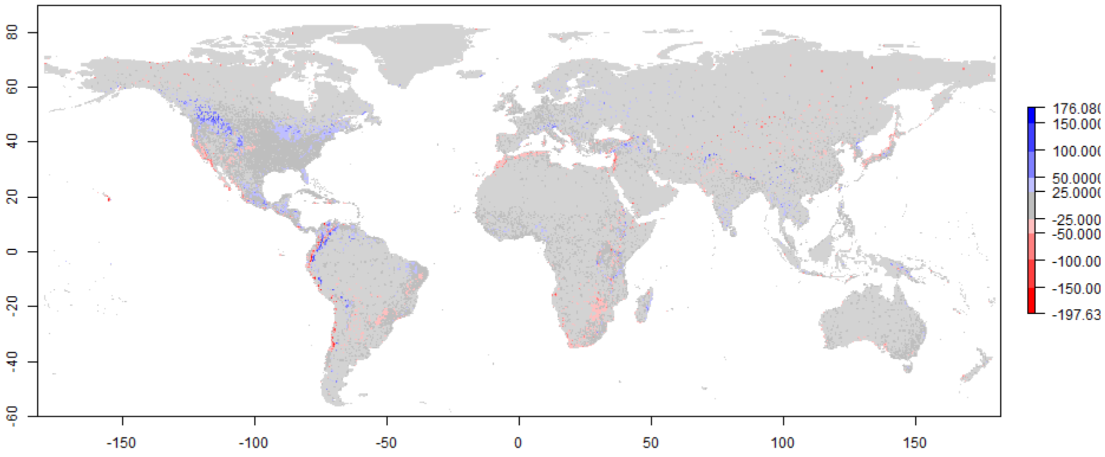

Supplementary Figure 5 (cont.)

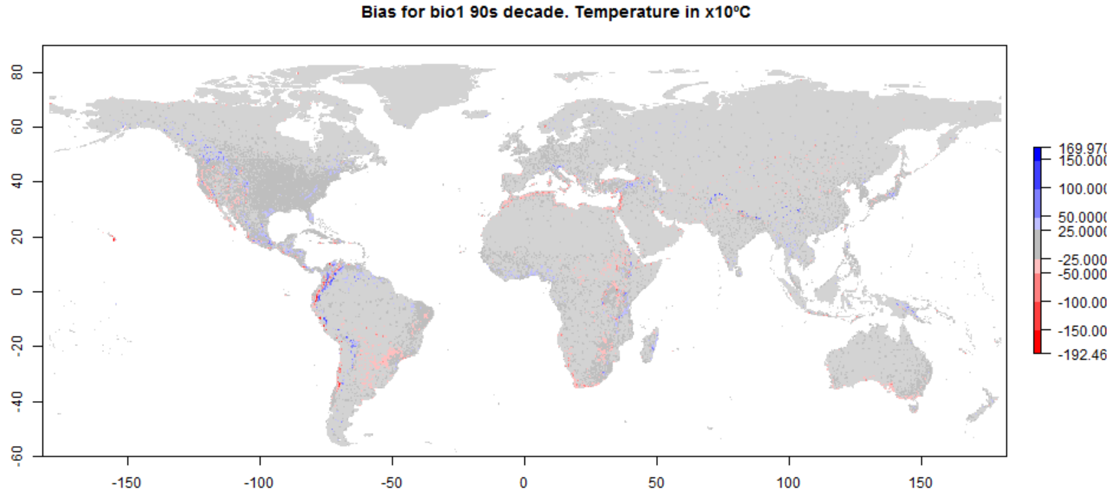

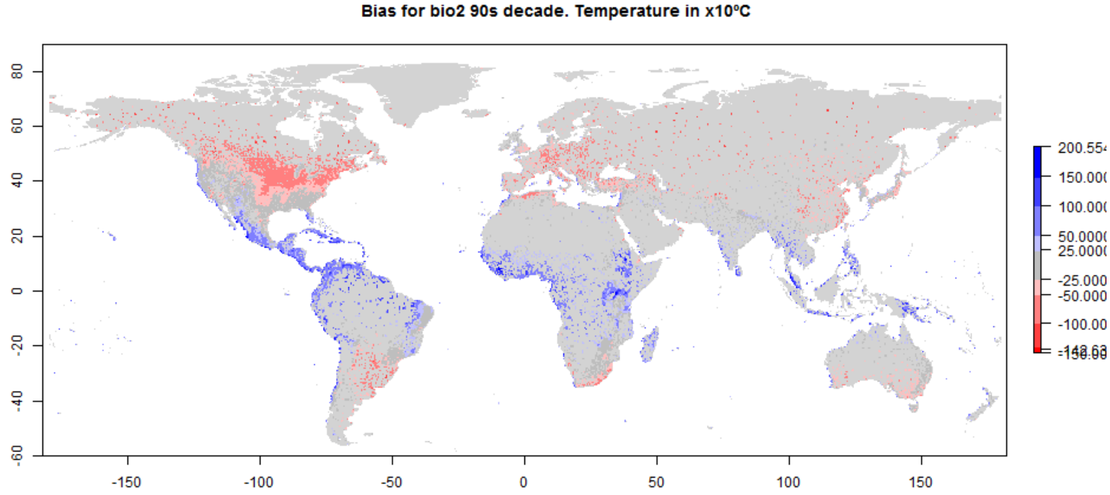

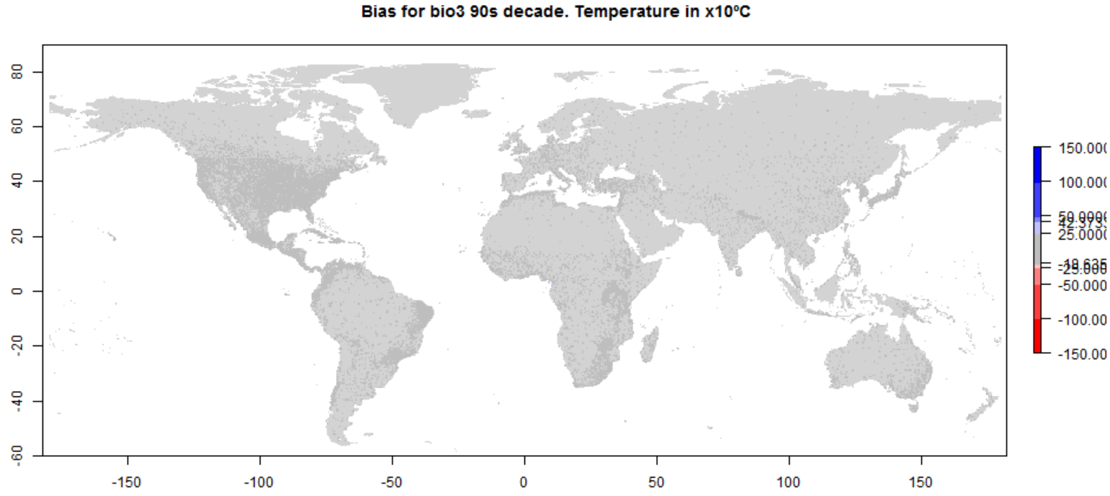

Supplementary Figure 5 (cont.)

Bias for bio4 90s decade. Temperature in x10ºC

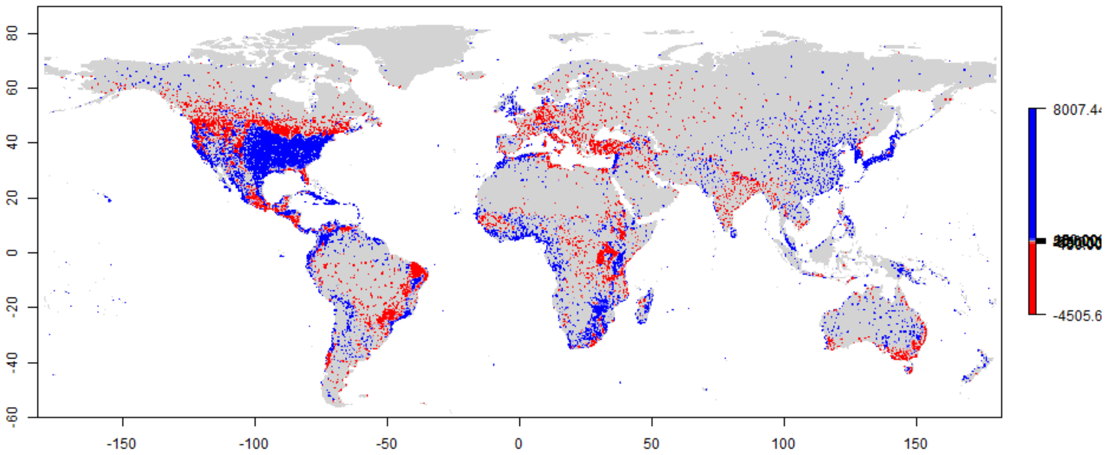

Bias for bio5 90s decade. Temperature in x10ºC

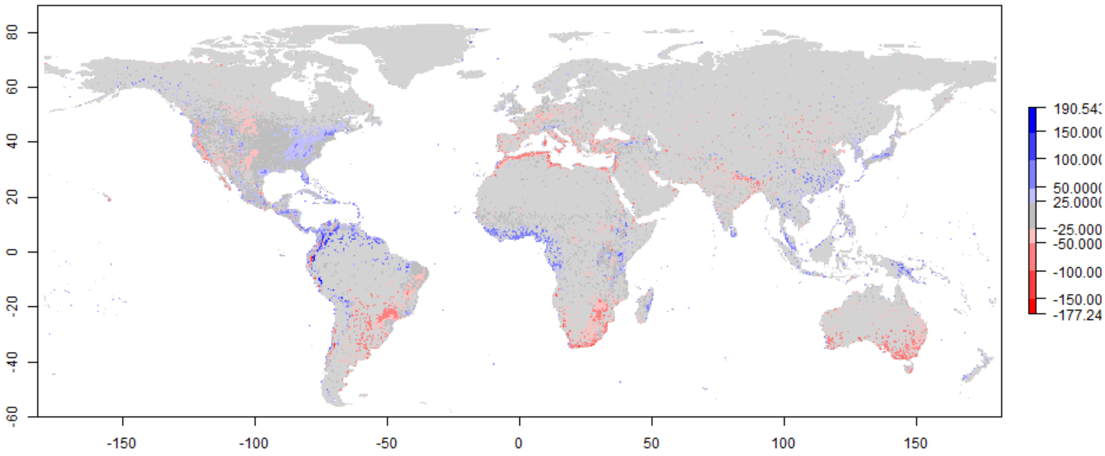

Bias for bio6 90s decade. Temperature in x10ºC

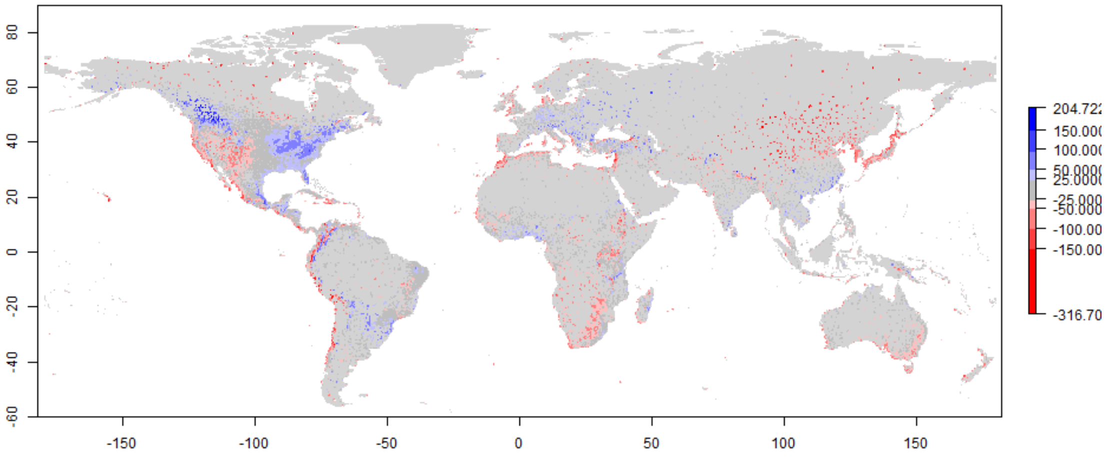

Supplementary Figure 5 (cont.)

Bias for bio7 90s decade. Temperature in x10ºC

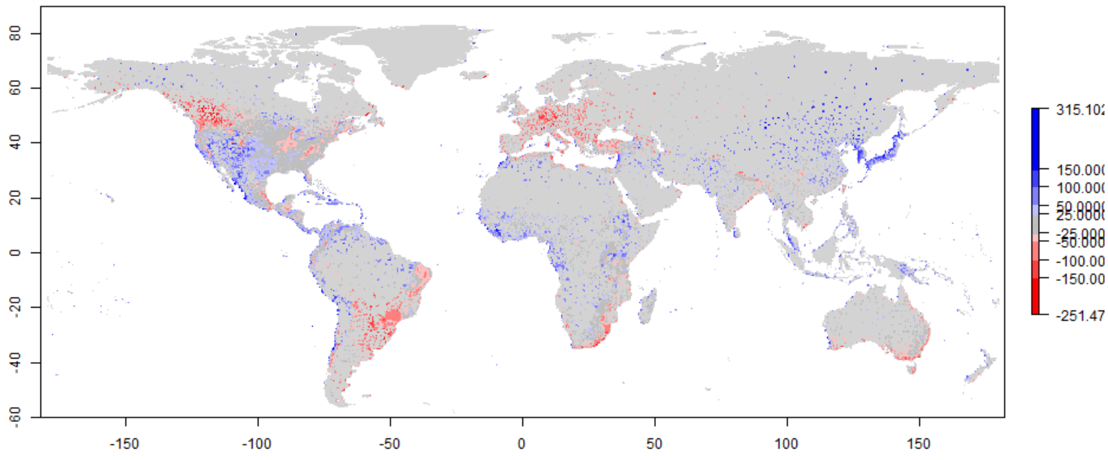

Bias for bio10 90s decade. Temperature in x10ºC

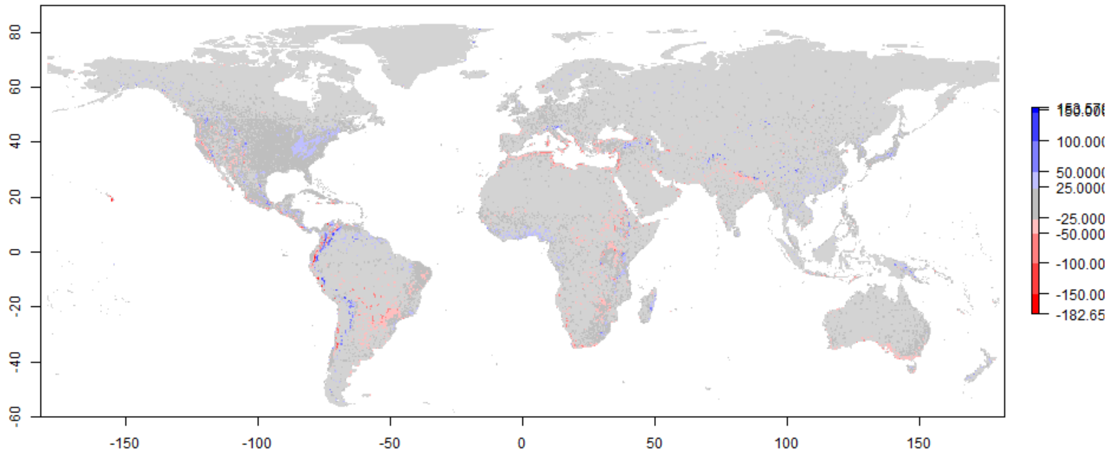

Bias for bio11 90s decade. Temperature in x10ºC

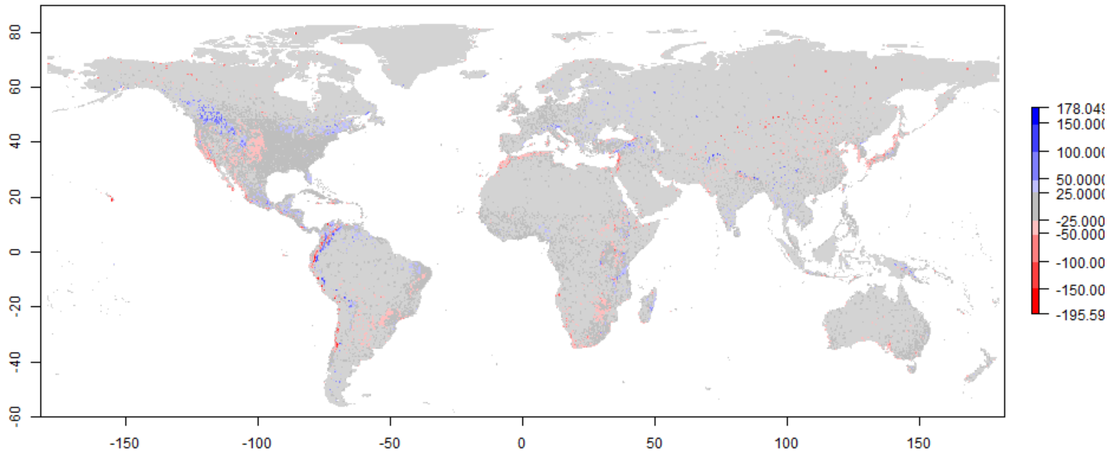

Supplement: Supplementary Information [file sdata201778-s1.pdf]
